# Hypoxia Induces Changes in AMP-Activated Protein Kinase Activity and Energy Metabolism in Muscle Tissue of the Oriental River Prawn *Macrobrachium nipponense*

**DOI:** 10.3389/fphys.2018.00751

**Published:** 2018-06-14

**Authors:** Shengming Sun, Zhongbao Gu, Hongtuo Fu, Jian Zhu, Xianping Ge, Xugan Wu

**Affiliations:** ^1^Key Laboratory of Genetic Breeding and Aquaculture Biology of Freshwater Fishes, Ministry of Agriculture, Freshwater Fisheries Research Centre, Chinese Academy of Fishery Sciences, Wuxi, China; ^2^Guangxi Academy of Fishery Sciences, Nanning, China; ^3^Key Laboratory of Exploration and Utilization of Aquatic Genetic Resources, Shanghai Ocean University, Ministry of Education, Shanghai, China

**Keywords:** *Macrobrachium nipponense*, AMPK, hypoxia, energy metabolism, aquaculture

## Abstract

Hypoxia has important effects on biological activity in crustaceans, and modulation of energy metabolism is a crucial aspect of crustaceans’ ability to respond to hypoxia. The adenosine 5′-monophosphate (AMP)-activated protein kinase (AMPK) enzyme is very important in cellular energy homeostasis; however, little information is known about the role of AMPK in the response of prawns to acute hypoxia. In the present study, three subunits of AMPK were cloned from the oriental river prawn, *Macrobrachium nipponense*. The full-length cDNAs of the α, β, and γ AMPK subunits were 1,837, 3,174, and 3,773 bp long, with open reading frames of 529, 289, and 961 amino acids, respectively. Primary amino acid sequence alignment of these three subunits revealed conserved similarity between the functional domains of the *M. nipponense* AMPK protein with AMPK proteins of other animals. The expression of the three AMPK subunits was higher in muscle tissue than in other tissues. Furthermore, the mRNA expression of AMPKα, AMPKβ, and AMPKγ were significantly up-regulated in *M. nipponense* muscle tissue after acute hypoxia. Probing with a phospho-AMPKα antibody revealed that AMPK is phosphorylated following hypoxia; this phosphorylation event was found to be essential for AMPK activation. Levels of glucose and lactic acid in hemolymph and muscle tissue were significantly changed over the course of hypoxia and recovery, indicating dynamic changes in energy metabolism in response to hypoxic stress. The activation of AMPK by hypoxic stress in *M. nipponense* was compared to levels of muscular AMP, ADP, and ATP, as determined by HPLC; it was found that activation of AMPK may not completely correlate with AMP:ATP ratios in prawns under hypoxic conditions. These findings confirm that the α, β, and γ subunits of the prawn AMPK protein are regulated at the transcriptional and protein levels during hypoxic stress to facilitate maintenance of energy homeostasis.

## Introduction

The concentration of dissolved oxygen is one of the most important factors influencing water quality in the growing industry of prawn aquaculture (Cheng et al., 2003), and can be a particularly limiting factor in rearing ponds that do not use aerators during hot summer weather. In crustaceans, hypoxia can increase lactate concentration and lipid peroxidation in tissues, while decreasing antioxidant capacity (Zenteno-Savín et al., 2006; Sun et al., 2014); this can result in tissue damage. Moreover, crustaceans can experience changes in immune response, behavior, reproduction, and biochemistry, depending on the severity and duration of hypoxic stress (McMahon, 2001; Craig et al., 2005; Brown-Peterson et al., 2008; Li and Brouwer, 2009, 2013; Paschke et al., 2009). Nevertheless, the impact of hypoxia on regulation of energy metabolism in crustaceans has not been extensively investigated, and it is of interest to elucidate the effects of hypoxia on energy metabolism in prawns.

Metabolic response is a common phenomenon in animals challenged by hypoxia. Some aquatic organisms switch to primarily utilize anaerobic metabolic pathways to supply energy in order to adapt to fluctuations of dissolved oxygen that occur during hypoxic stress (Harper and Reiber, 2006; Qiu, 2011). One key protein involved in regulating energy metabolism during hypoxia is adenosine 5′-monophosphate (AMP)-activated protein kinase (AMPK). AMPK is made up of three subunits; AMPKα, a catalytic subunit; AMPKβ, a scaffolding subunit; and AMPKγ, a regulatory subunit (Hardie and Carling, 1997).

Excluding lineage specific duplications of some AMPK subunits, the general AMPK structure is evolutionary conserved in animals; therefore, many characteristics of AMPK are common among different animal taxa (Craig et al., 2018). AMPK is important in stimulating glucose utilization in fish skeletal muscle (Magnoni et al., 2012); this suggested a possible role for AMPK in regulating hypoxia-induced energy responses in fish. In crustaceans, the major function of muscle tissue is to produce body movements, and the role of adipose tissue within muscle is to serve as a repository for energy storage and metabolism. We hypothesize that activation of AMPK under severe hypoxia exposure occurs in a tissue-specific manner, primarily occurring in muscle tissue.

Adenosine 5′-monophosphate (AMP)-activated protein kinase has been well-characterized in of a number of invertebrates, including the Atlantic rock crab, *Cancer irroratus* (Frederich et al., 2009), the American lobster, *Homarus americanus* (Jost et al., 2015), the green crab, *Carcinus maenas* (Toombs et al., 2011), the brine shrimp, *Artemia salina* (Zhu et al., 2007), the Pacific white shrimp, *Litopenaeus vannamei* (Xu et al., 2016), Pacific oyster *Crassostrea gigas* (Guévélou et al., 2013a), and the fruit fly *Drosophila melanogaster* (Yoshida et al., 1999). Additionally, depressed metabolic states have been associated with the AMPK response to hypoxia in insects (Hand et al., 2011). Nevertheless, the mechanisms regulating the activity of AMPK in crustaceans during hypoxia are poorly understood, particularly in commercial prawn species. The Oriental River Prawn, *Macrobrachium nipponense* (Crustacea; Decapoda; Palaemonidae) has a higher rate of oxygen consumption compared to other commercial shrimp species; this makes it an attractive model for hypoxia studies due to its susceptibility to hypoxic stress (Li et al., 2004). *M. nipponense* is an important species in Chinese aquaculture (Ma et al., 2011), and understanding the molecular mechanisms regulating hypoxic stress in *M. nipponense* is essential to the sustained development of Chinese prawn aquaculture.

In the present study, we describe the molecular cloning and sequencing of cDNAs that encode the α, β, and γ subunits of the AMPK protein from *M. nipponense* (referred to as MnAMPKα, MnAMPKβ, and MnAMPKγ). To investigate whether MnAMPK is involved in the molecular mechanisms of hypoxic stress in *M. nipponense*, we isolated full-length MnAMPK cDNA, and analyzed patterns of gene expression, activation, and enzyme activity in prawn muscle in conditions of hypoxia and normoxia. Furthermore, levels of energy metabolites, AMP, ADP, and ATP were measured in prawn muscle tissue in hypoxia vs. normoxia. The results of this study provide a foundation to investigate the AMPK signaling pathway in crustaceans.

## Materials and Methods

### Experimental Animals and Hypoxia Treatment

Healthy *M. nipponense* specimens (wet mass 2.28–3.74 g) were collected from Dapu experimental base near by Tai Lake, at the Freshwater Fisheries Research Centre of the Chinese Academy of Fishery Sciences (Wuxi, China). Prawns were acclimated in twelve 300 L aerated freshwater tanks for 2 weeks. Prawn aquaculture conditions were as follows: 24.2 ± 0.5°C, pH 8.3 ± 0.09, 6.8 ± 0.2 mg/L dissolved oxygen, with a natural photoperiod. For the hypoxia challenge experiment, prawns were divided into a control normoxia group and a hypoxia group; both groups were maintained in fresh filtered water. The control group was maintained under normoxia (6.5 ± 0.2 mg O_2_ L^-1^), and the hypoxia group was subjected to 2.0 ± 0.1 mg O_2_ L^-1^ for 1, 3, 6, 12, and 24 h by dissolving nitrogen into the tank, as previously described (Sun et al., 2014). All treatments were performed in triplicate for control and treatment groups. At each time point, muscle tissue was harvested from three prawns; tissue was immediately frozen in liquid nitrogen and stored at -80°C until further processing. This study was approved by the Institutional Animal Care and Use Ethics Committee of the Freshwater Fisheries Research Centre, Chinese Academy of Fishery Sciences (Wuxi, China).

### Cloning of the MnAMPKα, MnAMPKβ, and MnAMPKγ cDNAs

Total RNA was extracted from muscle tissue of prawns using TRIzol reagent, according to the manufacturer’s instructions. First strand cDNA synthesis was performed using a reverse transcriptase M-MLV kit (TaKaRa, Japan). Gene-specific primers (**Table 1**) were designed to the 5′- and 3′- ends of cDNA, based on the sequences of partial fragments, using 3′-RACE and 5′-RACE kits following the manufacturer’s instructions (TaKaRa). The partial cDNA sequences were obtained from an RNA-Seq database containing a transcriptome assembly of expressed short reads from *M. nipponense* (Sun et al., 2015). PCR products were sequenced on an ABI3730 DNA Analyzer after insertion into the pMD-19T vector.

**Table 1:** Primers used in this study.

Primer	Primer sequence (5′-3′)
MnAMPKα-F1 (5′ RACE out primer)	CTGGTTCCCAGACCATTACCC
MnAMPKα-F2 (5′ RACE in primer)	CACAGGGACAAAGGTAGCGAT
MnAMPKα-R1 (3′ RACE out primer)	AGGGCCTGCATAGAGTTTCC
MnAMPKα-R2 (3′ RACE in primer)	GAACCACAACTAGTGCGGAGA
MnAMPKβ-F1 (5′ RACE out primer)	CATTGCACCAACAGCACTCG
MnAMPKβ-F2 (5′ RACE out primer)	CAAGTGGACTGGAGGTGGTC
MnAMPKβ-R1 (3′ RACE out primer)	AGAACTGGAGGCCCTCGTAT
MnAMPKβ-R2 (3′ RACE in primer)	TGATGACCCTCGGGCAAATC
MnAMPKγ-F1 (5′ RACE out primer)	GGATTCCCACTCGGTGAAGG
MnAMPKγ-F2 (5′ RACE out primer)	CTAGCGACCACGGTAGTGAC
MnAMPKγ-R1 (3′ RACE out primer)	TGATGTGAGGAACCACTGCC
MnAMPKγ-R2 (3′ RACE in primer)	GCGTATTGTCCCCTGGACTC
MnAMPKα-F (Real-time primer)	TCACAGGGACAAAGGTAGCG
MnAMPKα-R (Real-time primer)	TCTGCGAGCTTCCGATTCTT
MnAMPKβ-F (Real-time primer)	CGGCCAGTCATAACACAGGG
MnAMPKβ-R (Real-time primer)	GCCCATGTTGTTGTCGCAAG
MnAMPKγ-F (Real-time primer)	CAGCTGGGAAAGCTTTTGGG
MnAMPKγ-R (Real-time primer)	GATGTGAGGAACCACTGCCA
β-Actin F (Real-time primer)	TATGCACTTCCTCATGCCATC
β-Actin R (Real-time primer)	AGGAGGCGGCAGTGGTCAT

### Nucleotide Sequence and Bioinformatics Analyses

Amino acid sequences were deduced using the ORF Finder program^1^. Sequences were analyzed using nucleotide and protein databases and employing the BLASTX and BLASTN programs^2^. Multiple sequence alignments of MnAMPKα, MnAMPKβ, and MnAMPKγ were carried out using the Clustal W Multiple Alignment program^3^. Phylogenetic trees were generated by the neighbor-joining method using MEGA software version 4.0^4^.

### qRT-PCR Analysis of MnAMPKα, MnAMPKβ, and MnAMPKγ Expression

Gene-specific primers (**Table 1**) were designed to analysis the expression of the three MnAMPK subunits in prawn tissue, including muscle, brain, gill, hepatopancreas, and hemocytes. cDNA was synthesized from tissues from the different treatment groups from total DNA-free RNA (1 μg) using a Prime Script RT reagent kit (TaKaRa), according to the manufacturer’s protocols. The stability of the reference genes was reported in our previous study (Sun et al., 2016). qRT-PCR was performed using a Bio-Rad iCycler iQ5 Real-Time PCR system (Bio-Rad, United States), and the reaction conditions have been previously described (Qiao et al., 2015). Briefly, the PCR temperature conditions were 95°C for 30 s followed by 40 cycles of 94°C for 15 s, 58°C for 20 s, and 72°C for 20 s, with a 0.5°C/5 s incremental increase from 60 to 95°C. Three replicate qPCR analyses were performed per sample, along with the internal control gene, and samples from three prawns were analyzed each time (*n* = 9). The expression levels of the MnAMPK subunits mRNAs were calculated using the 2^-ΔΔC_T_^ method (Livak and Schmittgen, 2001).

### Biochemical Assays

Frozen hemolymph and frozen prawn muscle tissue were thawed on ice (*n* = nine prawns per treatment group, representing three prawns per tank). Hemolymph glucose and lactic acid levels were assessed using a glucose assay kit and a lactic acid assay kit (F006 and A019-2, Nanjing Jiancheng Bioengineering Institute, China), respectively; each sample was analyzed in three technical replicates. Absorbance values at 505 and 530 nm were recorded and compared to the respective calibration curves to calculate glucose content and lactic acid content using spectrophotometric tests. The reaction products were evaluated using a microplate reader (BioTek, United States) and the results are presented according to the formulas provided with each protocol. Lactic acid and glycogen levels in muscle tissue were detected using a lactic acid assay kit and a glycogen assay kit (A019-2 and A403, Nanjing Jiancheng Bioengineering Institute), respectively. Muscle glycogen was determined according to the anthrone colorimetric method.

The activity of AMP-kinase in muscle was assayed by Shanghai Qiyi Biological Technology Co., Ltd.^5^ Tissue homogenates were prepared in phosphate-buffered saline (PBS, pH 7.4), according to an established protocol, and were centrifuged for 10 min at 5000 *g* to remove sediment. AMP-kinase activity was evaluated in the supernatant, according to the protocol provided with the ELISA kit [antibodies produced by Sangon Biotech (Shanghai) Co., Ltd.]. The results were quantified by a microplate reader, and activity was calculated following the formula provided in the protocol.

### Western Blotting

Muscle samples (∼20 mg) were homogenized in a buffer with an excess of phosphatase inhibitors to prevent dephosphorylation of AMPK. Total protein concentration was quantified using the Bradford (1976) method. An equal amount of protein from each sample (50 μg) was separated on a 10% SDS-polyacrylamide (SDS-PAGE) and transferred to a PVDF membrane (Millipore, Bedford, MA, United States) as previously described (Sun et al., 2017). Membranes were incubated with primary antibodies against AMPK and phosphorylated AMPK (Thr172) (1:1000; Cell Signaling Technology, Beverly, MA, United States) overnight at 4°C. Protein bands on membranes were visualized using a high sensitivity enhanced chemiluminescence kit, the ECL Advance^TM^ Western Blotting Detection Kit (GE Healthcare, Buckinghamshire, United Kingdom), according to the manufacturer’s instructions and then exposed to X-ray film. The X-ray films were developed, scanned, and the optical densities of the protein bands on the western blots were analyzed by densitometry using the computer-based Sigma Gel software, version 1.0 (Jandel Scientific, San Rafael, CA, United States). Protein amount was expressed relative to the amount of total protein homogenate loaded into each well, and normalized to the normoxic control samples.

### Determination of Adenine Nucleotide Levels

High-performance liquid chromatography (HPLC) has been widely employed as a method to analyze the levels of adenine nucleotide in various organisms. In this study, muscle tissue extracts were treated with perchloric acid and then filtered through a 0.45 μm HV-Millipore filter, and were then analyzed using an Agilent 1100 HPLC (Agilent Corp., United States) system. For HPLC separation, 20 μl aliquots of sample extract automatically injected onto an Ultimate^TM^ AQ-C18 column (4.6 mm × 250 mm) and adenylates were separated by the UV detector (254 nm) using phosphate buffer as the mobile phase. The flow rate was 1.0 ml⋅min^-1^ and column temperature was set at a constant 30°C. Sample peaks were calibrated and quantified with an HPLC chromatography data system. ATP, ADP, and AMP were identified based on comparison with retention time of known standards (Sangon, Shanghai, China), and the concentration of ATP, ADP, and AMP were determined using a method of external standards. Data are presented as mean ± SE, *n* = 9 for each group.

### Statistical Analysis

All data are presented as mean ± SE (standard error of the mean, *n* = 9). Student’s *t*-test was used to identify significant differences in expression of the three MnAMPK protein subunits between the control and test groups using SPSS 15.0 software. MnAMPK activity and tissue distribution were evaluated by one-way analysis of variance (ANOVA) using SPSS 15.0 software, with *post hoc* comparison of means using the Tukey–Kramer HSD test. A significance level of *P* ≤ 0.05 was considered significant. Data regarding AMPK expressions and biochemistry index were analyzed by two-way ANOVA for significant differences among treatment means based on sampling time, treatment type and their interaction. If significant differences were observed (*P* < 0.05) in the interaction, each factor was further analyzed separately by one-way ANOVA. Specifically, data among different sampling time within each treatment was analyzed by one-way ANOVA. Significant differences among groups were determined by Tukey’s HSD multiple range test.

## Results

### Cloning and Identification of MnAMPKα, MnAMPKβ, and MnAMPKγ

The prawn MnAMPKα transcript (KP050352) comprises 1,837 bp with start and stop codons at positions 124 and 123, respectively (**Supplementary Figure S1**). The deduced sequence of the MnAMPKα protein contained 529 amino acids, had an estimated molecular mass of 59.9 kDa, and an isoelectric point of 8.22. We identified a conserved activation loop fragment that includes a phosphorylation site (aa 161–184). This sequence also contained two domains that interact with the β and γ subunits, located at positions 412–525 and 456–518, respectively (**Supplementary Figure S4**). The MnAMPKβ cDNA sequence spans 3,174 bp, with start and stop codons at positions 38 and 907, respectively (**Supplementary Figure S2**). The MnAMPKβ sequence contains a conserved glycogen binding site in the 112–162 region (**Supplementary Figure S5**). In addition, we also characterized the prawn MnAMPKγ gene. The prawn MnAMPKγ cDNA sequence extends 3,773 bp, with start and stop codons at positions 55 and 2940, respectively (**Supplementary Figure S3**). MnAMPKγ has two conserved domains that are similar to domains of AMPKγ similar to those of other animals (Frederich et al., 2009; Jost et al., 2015). Two cystathionine beta-synthase domains were identified at positions 543–671 and 700–817 (**Supplementary Figure S6**). The GenBank accession numbers for MnAMPKβ and MnAMPKγ are MG792548 and MG792547, respectively. The deduced protein sequences of the MnAMPKβ and MnAMPKγ proteins were 290 and 996 amino acids in length, respectively. The estimated molecular masses of MnAMPKβ and MnAMPKγ were 33.08 and 106.79 kDa, respectively. MnAMPKβ was found to have an isoelectric point of 6.07, and the isoelectric point of MnAMPKγ was found to be 6.34.

### Phylogenetic Analysis of MnAMPKα, MnAMPKβ, and MnAMPKγ

**FIGURE 1:**
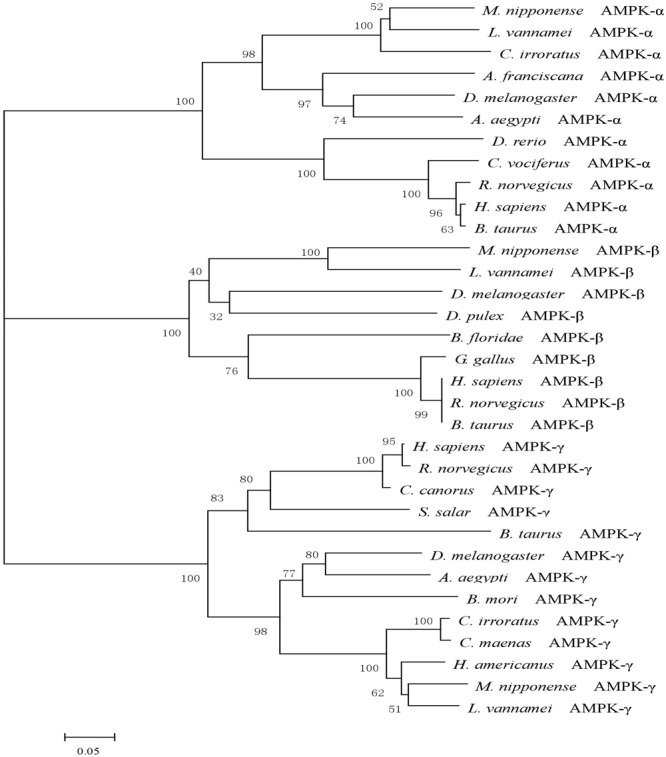
A phylogenetic tree of AMPK was constructed with neighbor-joining method by the program MEGA 6.0. The numbers at each branch indicate the percentage bootstrap values. The amino acid sequences were downloaded from NCBI: *Litopenaeus vannamei* (AKE50479.1), *Homo sapiens* (NP_006243.2), *Rattus norvegicus* (NP_076481.1), *Bos taurus* (NP_001192534.1), *Charadrius vociferous* (KGL92907.1), *Danio rerio* (NP_001103756.1), *Drosophila melanogaster* (NP_726730.1), *Aedes aegypti* (XP_001652572.1), *Cancer irroratus* (ACL13568.1), *Artemia franciscana* (ABI13783.1), *Litopenaeus vannamei* (AKE50480.1), *Homo sapiens* (NP_005390.1), *Rattus norvegicus* (NP_072149.1), *Bos taurus* (NP_001179257.1), *Gallus gallus* (NP_001038127.1), *Branchiostoma floridae* (EEN66070.1), *Drosophila melanogaster* (NP_610460.1), *Daphnia pulex* (EFX67948.1), *Litopenaeus vannamei* (AKE50481.1), *Homo sapiens* (NP_001035723.1), *Rattus norvegicus* (NP_908940.1), *Bos taurus* (NP_001025473.2), *Cuculus canorus* (KFO74243.1), *Bombyx mori* (NP_001119720.1), *Salmo salar* (ACI33670.1), *Drosophila melanogaster* (NP_732599.1), *Aedes aegypti* (XP_001659398.1), *Cancer irroratus* (ACL13567.1), *Homarus americanus* (AEO22037.1), and *Carcinus maenas* (AEO22038.1).

A phylogenetic tree was constructed from the amino acid sequences of the AMPK protein from eight different species (**Figure 1**). The AMPKα, AMPKβ, and AMPKγ subunits of the oriental river prawn (*M. nipponense*) showed close phylogenetic similarity with those of the Pacific white shrimp (*Litopenaeus vannamei*).

### Tissue-Specific mRNA Expression of MnAMPKα, MnAMPKβ, and MnAMPKγ

**FIGURE 2:**
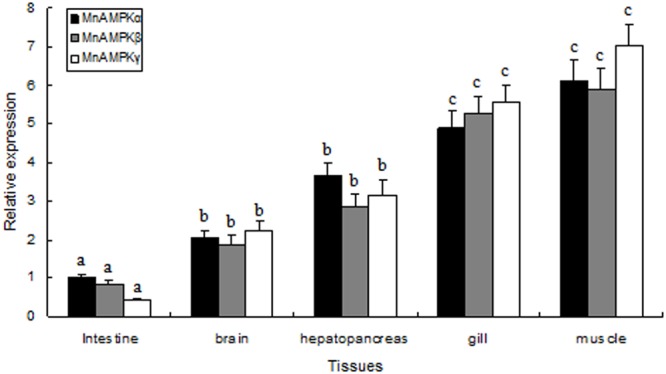
Distribution of mRNA expression of the three AMPK subunits, α, β, and γ, in different tissues of *M. nipponense*, as assessed by real-time quantitative RT-PCR. MnAMPKα expression in intestine was used as a reference for relative expression. Values are expressed as the mean ± SE (*n* = 9). Significant differences (*P* < 0.05) among all tissues are indicated by different letters for each AMPK subunits.

The distribution of MnAMPKα, MnAMPKβ, and MnAMPKγ expression in different prawn tissues was examined by qRT-PCR. Expression of the MnAMPKα, MnAMPKβ, and MnAMPKγ transcripts were broadly expressed across many prawn tissues, including intestine, gill, brain, muscle, and hepatopancreas (**Figure 2**). MnAMPKα, MnAMPKβ, and MnAMPKγ mRNAs were highly expressed in muscle and hepatopancreas, while significantly lower expression levels were found in intestine tissue (*P* < 0.05).

### Expression of MnAMPKα, MnAMPKβ, and MnAMPKγ During Hypoxia

**FIGURE 3:**
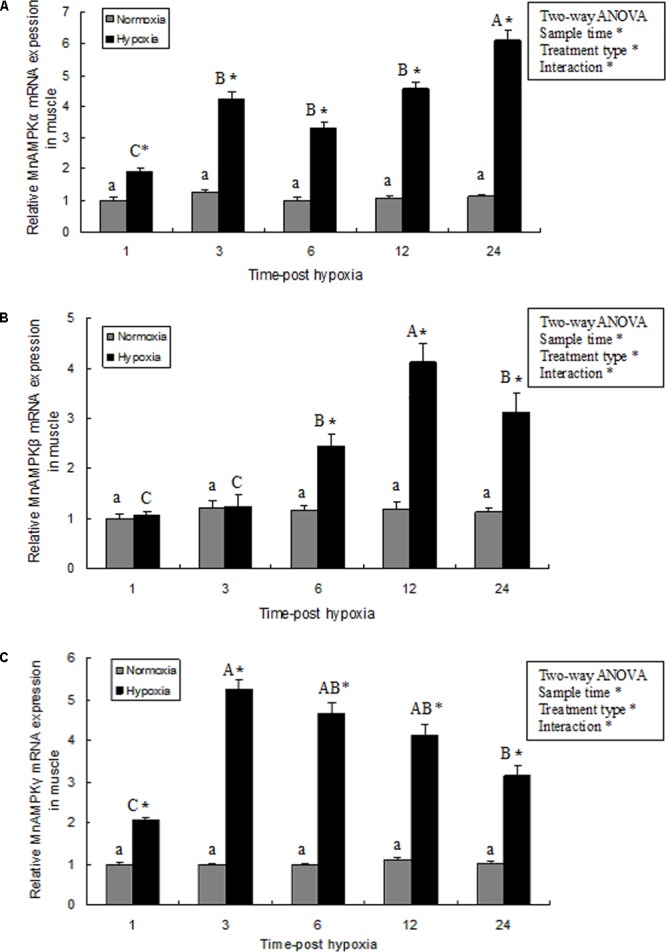
The effect of hypoxia on AMPKα **(A)**, AMPKβ **(B)**, and AMPKγ **(C)** mRNA expression levels in muscle tissue in *M. nipponense*. mRNA expression of the AMPK subunits (α, β, and γ) was normalized to the expression level in the normoxia group. Values are expressed as the mean ± SE (*n* = 9). Significant differences (*P* < 0.05) among sampling times within each treatment are indicated by different letters (upper case for hypoxia, and lower case for mormoxia). Student’s *t*-test was used to compare expression between the hypoxia and control groups. Error bars indicate the standard error and asterisks indicate significant differences between the hypoxia and normoxia groups (*P* < 0.05).

In muscle tissue, MnAMPK expression was significantly affected by sampling time (*P* < 0.05), treatment type (*P* < 0.001), and their interaction (*P* < 0.05). However, the MnAMPK expression of the normoxia treatment showed no statistical difference (*P* > 0.05) during the whole sampling period. In terms of treatment type, the MnAMPK expression of prawns was significantly (*P* < 0.001) higher than that of the normoxia treatment. Furthermore, the expression of MnAMPKα was significantly upregulated (*P* < 0.05) after 3 h hypoxia compared to normoxia. Subsequently, the expression of AMPKα increased at 6 and 12 h, and then reached a maximum point (**Figure 3A**). The pattern of expression of MnAMPKβ after exposure to hypoxia was similar to that of MnAMPKα (**Figure 3B**), except expression of MnAMPKβ began to significantly increase after 6 h of hypoxia. Similarly, the expression patterns of MnAMPKγ corresponded to that of MnAMPKα and MnAMPKβ, reaching a maximum at 12 h of hypoxia exposure (**Figure 3C**).

### Activation and Activity of AMPK in Prawn Muscle Tissue in Response to Hypoxia

**FIGURE 4:**
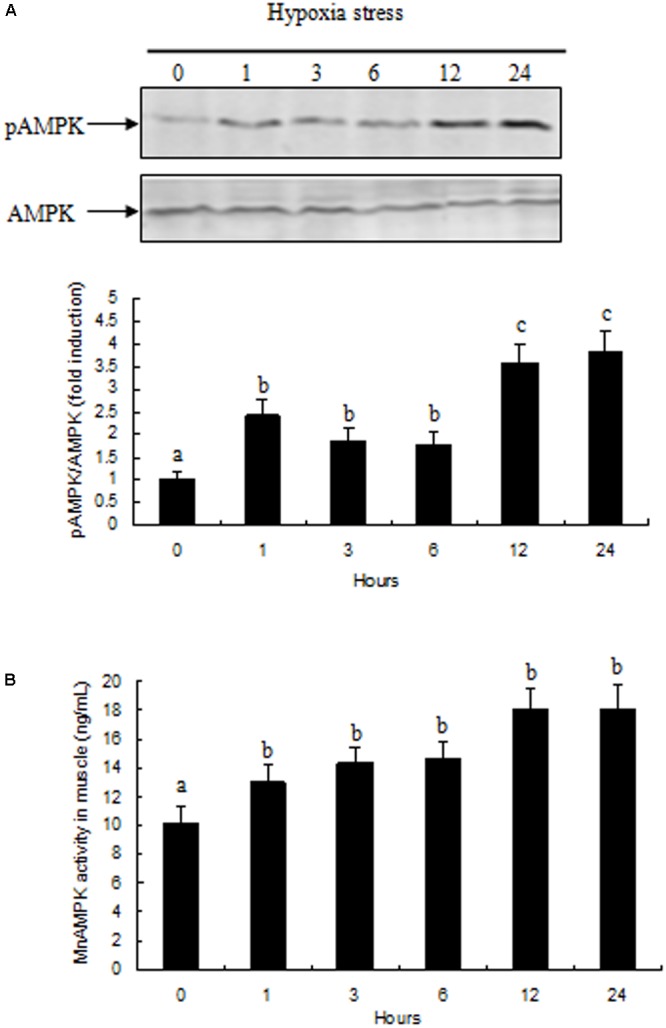
Effects of hypoxia on AMPK activity in muscle of *M. nipponense*. **(A)** pAMPK/AMPK abundance are represented as a fold induction over basal levels observed in the normoxia group at time 0 h. **(B)** Changes in AMPK enzyme activity in response to hypoxia in muscle of *M. nipponense*. Results are expressed as mean ± SE (*n* = 9). Different letters indicate significant differences among all treatment groups (*P* < 0.05).

Activation of AMPK in prawn muscle tissue was assessed by evaluating the ratio between pAMPK and total AMPK. Activation of AMPK in prawn muscle significantly increased during hypoxia (**Figure 4A**). AMPK activity in muscle tissue exhibited a similar pattern with the changes observed in gene expression, and increased rapidly after exposure to 1 and 3 h of hypoxia (*P* < 0.05 compared to normoxia group); moreover, the activity of AMPK activity in samples at hypoxia 12 and 24 h were both significantly increased (*P* < 0.05) compared with other treatment groups (**Figure 4B**).

### Energy Metabolic Response After Acute Hypoxic Stress

**FIGURE 5:**
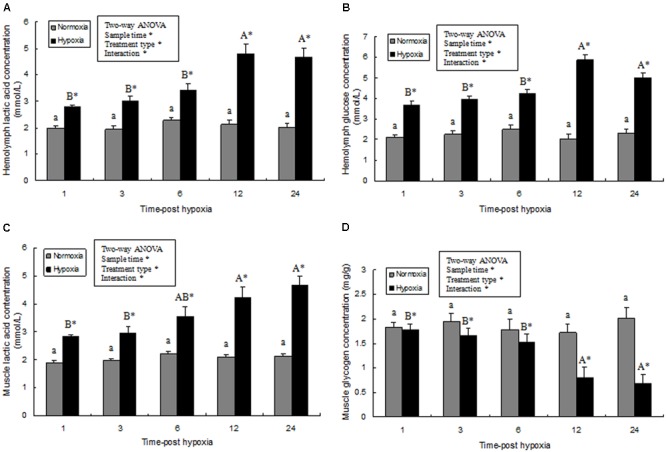
Effects of hypoxia on hemolymph lactic acid **(A)**, hemolymph glucose concentration **(B)**, muscle lactic acid levels **(C)**, and muscle glycogen content **(D)**, respectively. Results are expressed as mean ± SE (*n* = 9). Significant differences (*P* < 0.05) among sampling times within each treatment are indicated by different letters (upper case for hypoxia, and lower case for mormoxia). Student’s *t*-test was used to compare the hypoxia and control groups. Asterisks indicate significant differences between the hypoxia group and normoxia group (*P* < 0.05).

Hemolymph glucose and lactic acid contents and the glycogen content of prawn muscle tissue was significantly affected by sampling time (*P* < 0.05), treatment type (*P* < 0.001), and their interaction (*P* < 0.05). However, the glucose and lactic acid contents and the glycogen content of the prawns in normoxia treatment showed no statistical difference (*P* > 0.05) during the whole sampling period. Further, hemolymph glucose and lactic acid contents significantly increased after exposure to hypoxia for 3 h (*P* < 0.05), and continued to increase significantly (**Figures 5A,B**). At 24 h, the lactic acid concentration in the muscle tissue of the hypoxia group was significantly higher than that of the control group (**Figure 5C**; *P* < 0.05). On the contrary, the glycogen content in muscle tissue from the hypoxia group was lower than that in the control group at 12–24 h (**Figure 5D**; *P* < 0.05).

### Adenine Nucleotides in Prawn Muscle Tissue

**FIGURE 6:**
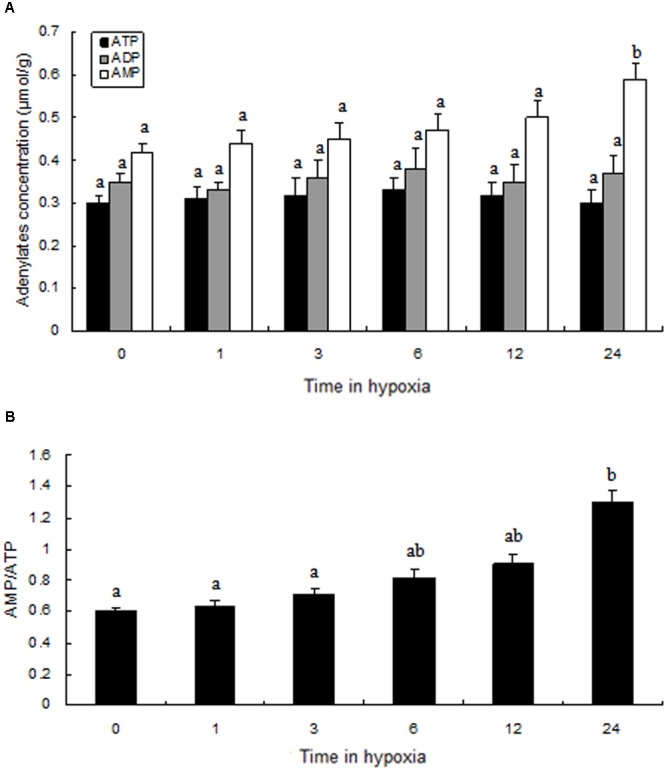
Levels of adenylates in the muscle of prawns during hypoxia exposure. **(A)** Adenylate concentrations; **(B)** the ratio of AMP to ATP. Results are expressed as mean ± SE (*n* = 9). Different letters indicate significant differences among all treatment groups (*P* < 0.05).

Compared to the levels of the control group, ATP, ADP, and AMP levels changed slightly during hypoxic stress (**Figure 6A**). We found that, although there was not a significant difference in ATP and ADP values between the normoxia and hypoxia groups, the AMP levels in the hypoxia group after 24 h were significantly higher than in the control group. We observed a slow increase in AMP:ATP ratio following hypoxia, and a significantly higher AMP:ATP ratio was observed in the hypoxia group after 24 h (**Figure 6B**).

## Discussion

In terrestrial vertebrates, AMP-activated protein kinase (AMPK) acts as a sensor for intracellular energy, and regulates energy metabolism (Hardie, 2003). On the contrary, less is known about the function of AMPK in energy metabolism of aquatic species, especially freshwater prawns. In this study, we identify the full sequences of the α, β, and γ subunits that constitute the AMPK protein of the Oriental river prawn, *M. nipponense*. The sequence of the MnAMPKα subunit displayed high identity to the AMPKα subunit of other species, and contained several conserved functional sites (Littler et al., 2010), including a phosphorylation site (α-Thr172) that acts as a major regulatory element, located in the AMPKα activation loop (Beauloye et al., 2001; Horman et al., 2006); this site is critical for phosphorylation of AMPKα by its upstream kinase, AMPK (Hardie, 2008). The activation loop of MnAMPKα contains a highly conserved activation loop, with an amino acid sequence of ADFGLSNMMVDGEFLRTSCGSPN, demonstrating the conservation of AMPKα throughout animal evolution.

AMPKβ acts as a scaffold to assemble the AMPKα and AMPKγ subunits; AMPKβ also can regulate the substrate specificity and subcellular localization of the AMPK complex (Rubio et al., 2013). Compared to the MnAMPKα subunit, the deduced amino acid sequence of MnAMPKβ has lower sequence identity to the AMPKβ subunits of other animals. AMPKβ contains an evolutionarily conserved carbohydrate binding motif that binds with oligosaccharides and inhibits AMPK T172 phosphorylation (McBride et al., 2009). We identified a glycogen-binding domain in MnAMPKβ that has a slightly different amino acid sequence compared to AMPKβ from other animals (Colbourne et al., 2011); the mechanism of this glycogen-binding domain in AMPKβ of aquatic animals is poorly understood.

Unlike the AMPKα and AMPKβ subunits, little is known about the MnAMPKγ gene, and the nucleotide sequences and sequences of the AMPKγ full-length cDNA reported in NCBI show broad variation. These differences suggest that the function of MnAMPKγ may not have been conserved over the course of evolution; nevertheless, both of the CBS-pairs in MnAMPKγ are highly conserved, and both are critical for the mutually exclusive binding of AMP or ATP (Scott et al., 2004). Phylogenetically, the α, β, and γ subunits of *M. nipponense* AMPK cluster in a monophyletic group with their orthologous counterparts. Multiple sequence alignment, amino acid structural analysis, and phylogenetic tree construction confirmed that the cDNAs cloned in this study were indeed the α, β, and γ subunits of *M. nipponense* AMPK.

In this study, we found that the expression levels of the three MnAMPK subunits were higher in gills and muscle tissue than in the intestine. The elevated expression of the three MnAMPK subunits in the muscle and gill are consistent with higher energy metabolism in muscle tissue and higher energy consumption in gill tissue (Li et al., 2007). Hepatopancreas is capable of adjusting metabolism after a short response period, and it has been proposed that high abundance of AMPK gene transcripts are involved in hepatopancreas function. The lower expression of the three MnAMPK subunits in the intestine may be related with the physiological function of this organ. The intestine produces a large amount of digestive enzymes (Muhlia-Almazán et al., 2003), and the relative abundance of transcripts of genes involved in other central pathways, such as AMPK, may be lower; the basis for this observation requires further investigation.

We observed changes in gene expression of the three MnAMPK subunits under conditions of acute hypoxic stress. One hour after hypoxic stress, MnAMPKα mRNA expression was significantly increased in prawn muscle tissue; this is consistent with previous studies where hypoxic stress altered AMPK mRNA expression and activity in vertebrates and invertebrates, including rat (Fukuyama et al., 2007), human (Mungai et al., 2011), mice (Rousset et al., 2015), and oyster (Guévélou et al., 2013b). These studies indicate that transcriptional regulation of AMPKα (Hawley et al., 1996) may be involved in regulating energy metabolism in response to acute hypoxic stress in aquatic animals. We also found that hypoxia induced phosphorylation of AMPK, indicating that AMPK function and activity may be related to hypoxia. Previous studies demonstrated that AMPKβ is closely related to carbohydrate metabolism in mice and fish (Steinberg et al., 2010; Magnoni et al., 2012); our present study found that expression of MnAMPKβ was induced in prawns after 6 h of hypoxic stress; this occurred along with significant changes in muscular carbohydrate metabolism. MnAMPKγ mRNA levels in the muscle tissue of prawns in the hypoxia group were significantly higher than those of the control group; this is consistent with a previous study in the rock crab *Cancer irroratus* (Frederich et al., 2006). AMPK enzyme activity is also significantly induced by hypoxic stress to regulate rapid energy consumption; this appears to be coordinated by the activation of AMPK.

**FIGURE 7:**
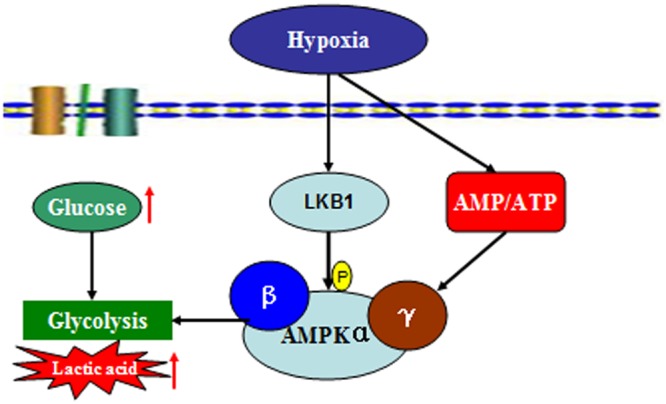
Summary of the molecular signaling events and cellular responses related to energy metabolism and AMPK activation in the muscle tissue of *M. nipponense* under hypoxic stress.

To date, this is the first study to explore changes in AMPK activation in prawns under hypoxic stress. We observed a slow increase in the AMP:ATP ratio in prawn muscle tissue in response to hypoxia; it has been suggested that this contributes to the maintenance of a stable cellular ATP supply in prawns during exposure to hypoxia. However, the concentration of cellular free ADP ([ADP free]) and free AMP ([AMP free]) in prawn muscle tissue under hypoxic conditions needs to be further studied in order to ascertain if regulation of AMPK relates to changes in cellular [AMP free]:[ATP] (Jibb and Richards, 2008). Upregulation of the AMP:ATP ratio cannot solely explain AMPK activation. We have previously demonstrated that LKB1 mRNA expression levels in prawns are sensitive to hypoxia, follow consistent patterns with AMPK phosphorylation levels (unpublished). AMPK activation in *M. nipponense* coincides with the expression of LKB1, suggesting that AMPK may be primarily regulated by LKB1, demonstrating AMPK regulation that is independent of adenosine nucleotide levels (Evans et al., 2012). Thus, we speculate that LKB1 may be a critical kinase upstream of AMPK in prawns under acute hypoxic stress (**Figure 7**).

In order to describe the mechanism by which activated-AMPK regulates energy balance in muscle tissue by accelerated ATP generation pathways, we analyzed the energy metabolic response of prawns after acute hypoxia stress. We found that muscle glycogen was substantially decreased following hypoxic stress, while lactic acid was increased in muscle tissue. It is possible that metabolism of muscle glycogen contributed significantly to total energy consumption in conditions of acute hypoxia; this is consistent with previous studies in *Palaemon elegans* and *P. serratus* (Taylor and Spicer, 1987). Glucose and lactic acid levels in hemolymph were significantly higher in prawns under hypoxic conditions than in the control group, indicating *M. nipponense* stress status and suggesting that carbohydrates are consumed as fuel during hypoxia in *M. nipponense*; this is in agreement with studies from *Neotrypaea uncinata* and *Litopenaeus vannamei* (Soñanez-Organis et al., 2009, 2010; Leiva et al., 2015).

## Conclusion

In conclusion, here we describe the full-length cDNA sequences encoding the three AMPK subunits of *M. nipponense* (AMPKα, β, and γ). We report that the transcriptional regulation, phosphorylation, and enzyme activity of AMPK are induced by hypoxia in *M. nipponense*; this is consistent with our previous observations that LKB1 mRNA expression levels are induced by hypoxia. We also observed changes in energy metabolism in muscle tissue when prawns were exposed to hypoxia. These data demonstrate that phospho-AMPK is primarily regulated by LKB1, and plays a critical role in maintaining energy balance in prawn muscle tissue under conditions of hypoxic stress.

## Author Contributions

SS, ZG, and HF conceived and designed the experiments. SS, XW, JZ, and ZG carried out the experiments and analyzed the data. SS, HF, and XG supervised the project. SS wrote the manuscript. All authors reviewed the manuscript.

## Conflict of Interest Statement

The authors declare that the research was conducted in the absence of any commercial or financial relationships that could be construed as a potential conflict of interest.

## Footnotes

1 http://ncbi.nlm.nih.gov/gorf/gorf.html

2 http://www.ncbi.nlm.nih.gov/BLAST/

3 http://www.ebi.ac.uk/clustalw/

4 http://www.megasoftware.net/mega4/mega.html

5 http://www.qiyibio.com/

## Supplementary Material

FIGURE S1 The full-length cDNA of the AMPKα subunit gene of the oriental river prawn and the deduced amino acid sequence are shown. Uppercase letters indicate the translated region, lowercase letters indicate the untranslated region, and the asterisk indicates the stop codon. The protein kinase ATP-binding region signature is shaded, the serine/threonine protein kinase active-site signature is underlined, and the activation loop region signature is boxed.

FIGURE S2 The full-length cDNA of the AMPKβ subunit gene of the oriental river prawn and the deduced amino acid sequence are shown. Uppercase letters indicate the translated region, lowercase letters indicate the untranslated region, and the asterisk indicates the stop codon. The glycogen binding site is shaded.

FIGURE S3 The full-length cDNA of the AMPKγ subunit gene of the oriental river prawn and the deduced amino acid sequence are shown. Uppercase letters indicate the translated region, lowercase letters indicate the untranslated region, and the asterisk indicates the stop codon. Cystathionine beta-synthase (CBS pair) domains are underlined.

FIGURE S4 Amino acid alignment of AMPKα from *M. nipponense* with ten other known sequences of the AMPKα subunit: *Litopenaeus vannamei* (AKE50479.1), *Homo sapiens* (NP_006243.2), *Rattus norvegicus* (NP_076481.1), *Bos taurus* (NP_001192534.1), *Charadrius vociferous* (KGL92907.1), *Danio rerio* (NP_001103756.1), *Drosophila melanogaster* (NP_726730.1), *Aedes aegypti* (XP_001652572.1), *Cancer irroratus* (ACL13568.1), and *Artemia franciscana* (ABI13783.1).

FIGURE S5 Amino acid alignment of AMPKβ from *M. nipponense* with eight other known sequences of the AMPKβ subunit: *Litopenaeus vannamei* (AKE50480.1), *Homo sapiens* (NP_005390.1), *Rattus norvegicus* (NP_072149.1), *Bos taurus* (NP_001179257.1), *Gallus gallus* (NP_001038127.1), *Branchiostoma floridae* (EEN66070.1), *Drosophila melanogaster* (NP_610460.1), and *Daphnia pulex* (EFX67948.1).

FIGURE S6 Amino acid alignment of AMPKγ from *M. nipponense* with twelve other known sequences of the AMPKγ subunit: *Litopenaeus vannamei* (AKE50481.1), *Homo sapiens* (NP_001035723.1), *Rattus norvegicus* (NP_908940.1), *Bos taurus* (NP_001025473.2), *Cuculus canorus* (KFO74243.1), *Bombyx mori* (NP_001119720.1), *Salmo salar* (ACI33670.1), *Drosophila melanogaster* (NP_732599.1), *Aedes aegypti* (XP_001659398.1), *Cancer irroratus* (ACL13567.1), *Homarus americanus* (AEO22037.1), and *Carcinus maenas* (AEO22038.1).
