# Supplementary material for: Hypoxia Induces Changes in AMP-Activated Protein Kinase Activity and Energy Metabolism in Muscle Tissue of the Oriental River Prawn Macrobrachium nipponense
Source: Front Physiol. 2018 Jun 14;9:751. doi: 10.3389/fphys.2018.00751 (PMC6011032; doi:10.3389/fphys.2018.00751)
Supplement: FIGURE S1 — The full-length cDNA of the AMPKα subunit gene of the oriental river prawn and the deduced amino acid sequence are shown. Uppercase letters indicate the translated region, lowercase letters indicate the untranslated region, and the asterisk indicates the stop codon. The protein kinase ATP-binding region signature is shaded, the serine/threonine protein kinase active-site signature is underlined, and the activation loop region signature is boxed. [file Data_Sheet_1.PDF]

Figure S1

```
       cgggtctgtccctgaatagggaagagccttcggaaggactctgctgccagattacaaatt
       gtaaacaagtggtgaactacagtgatatatcttccctactaaattactagaacacacggc
-124   tatcATGGAGTTGGGTCAAGGACAAGGACCTGGTTCCCAGACCATTACCCTAATGAAAATT
1          M  E  L  G  Q  G  Q  G  P  G  S  Q  T  I  T  L  M  K  I
58     GGTCATTATCAGATTGGAAATACGCTAGGATCAGGAACTTTTGGCAAAGTCAAATATGGA
20      G  H  Y  Q  I  G  N  T  L  G  S  G  T  F  G  K  V  K  Y  G
118    GAGCATATCCTCACAGGGACAAAGGTAGCGATTAAGATCCTCAACAGAAGGACCATAAAA
40      E  H  I  L  T  G  T  K  V  A  I  K  I  L  N  R  R  T  I  K
178    AATTTAGATATGGTCAGTAAAATCAAAAGGGAGATAACTAATCTCAAATTGTTTCGCCAT
60      N  L  D  M  V  S  K  I  K  R  E  I  T  N  L  K  L  F  R  H
238    CCACATATCATTAAGCTCTATCAAGTAATTAGTACTCCAACGGACATTTTCATGGTAATG
80      P  H  I  I  K  L  Y  Q  V  I  S  T  P  T  D  I  F  M  V  M
298    GAGTATGCATCTGGAGGTGAATTGTTTGATTACATTAAAGAGAAAGGAAAGTTGAAAGAA
100     E  Y  A  S  G  G  E  L  F  D  Y  I  K  E  K  G  K  L  K  E
358    TCGGAAGCTCGCAGATTTTTCCAGCAAATTATATCTGGTGTAGATTACTGCCACCGTCAC
120     S  E  A  R  R  F  F  Q  Q  I  I  S  G  V  D  Y  C  H  R  H
418    ATGGTTGTACATAGGGATTTAAAGCCAGAAAACCTGCTCCTTGACCATAACCTTCATGTC
140     M  V  V  H  R  D  L  K  P  E  N  L  L  L  D  H  N  L  H  V
478    AAAATTGCAGACTTTGGTCTGTCAAATATGATGGTAGATGGTGAATTTCTCCGCACTAGT
160     K  I  A  D  F  G  L  S  N  M  M  V  D  G  E  F  L  R  T  S
538    TGTGGTTCTCCGAATTATGCTGCCCCAGAAGTCATTTCTGGGAAACTCTATGCAGGCCCT
180     C  G  S  P  N  Y  A  A  P  E  V  I  S  G  K  L  Y  A  G  P
598    GAGGTTGATGTTTGGTCTTGTGGCATTATCTTGTATGCTCTCCTTTGTGGTACACTGCCA
200     E  V  D  V  W  S  C  G  I  I  L  Y  A  L  L  C  G  T  L  P
658    TTTGATGATGAACATGTACCATCGCTTTTCAAAAAAATCAAGTCTGGTGTGTTCCAGATT
220     F  D  D  E  H  V  P  S  L  F  K  K  I  K  S  G  V  F  Q  I
718    CCTGATTATTTAAATCAGAGTGTAGTACGTCTCCTCCTCCACATGCTTATGGTAGATCCA
240     P  D  Y  L  N  Q  S  V  V  R  L  L  L  H  M  L  M  V  D  P
778    ATGAAAAGAGCCACAGTTGATGATATTAAGAAGCATGAGTGGTTTCAAAGAGATTTGCCT
260     M  K  R  A  T  V  D  D  I  K  K  H  E  W  F  Q  R  D  L  P
838    GCATATCTTTTCCCTCCTCCTTATGATCTTGATAGTTCAGTTATTGACCAAGATGCTATC
280     A  Y  L  F  P  P  P  Y  D  L  D  S  S  V  I  D  Q  D  A  I
898    AGTGAAGTTTGCGAGAAATTCCAAGTGGAAACTTCAGAAGTACAGGATGCCCTTTTGTCA
300     S  E  V  C  E  K  F  Q  V  E  T  S  E  V  Q  D  A  L  L  S
958    GAAGACCCACACAATCAACTGAAGATTGCCTACAATCTTATTGTTGATAATAAGAGATTT
320     E  D  P  H  N  Q  L  K  I  A  Y  N  L  I  V  D  N  K  R  F
1018   GCTGATGCTAATGCACTGTATAGTATATCTGCTTTCTATTCAGCTGGATCTCCACCACCA
340     A  D  A  N  A  L  Y  S  I  S  A  F  Y  S  A  G  S  P  P  P
1078   AATAATCCACCAACACCAGCTTTCAGCCCACTGGATTCAAGTCCGAGTAGCTGCAAGCCT
360     N  N  P  P  T  P  A  F  S  P  L  D  S  S  P  S  S  C  K  P
1138   CATCCTGAGCGTATAGCGCGTAAGTCCAAAAATGCTTTGCGTGAAAGGGCCCTAAGTGGA
380     H  P  E  R  I  A  R  K  S  K  N  A  L  R  E  R  A  L  S  G
1198   GACAGAGGAGTACCAAAAGGAACGCCAGTTAAGAGAGCAAAGTGGCATCTTGGTATCCGT
```

```
400     D  R  G  V  P  K  G  T  P  V  K  R  A  K  W  H  L  G  I  R
1258   TCTCAGAGCAAACCACTTGACATAATGAGTGAAGTCTACAAAGCCATGAAGGTTCTTGGA
420     S  Q  S  K  P  L  D  I  M  S  E  V  Y  K  A  M  K  V  L  G
1318   TTTGAGTGGAAGGTCGTTAACCCTTTCCATGTGCGAGTTCGACGAAAGAACCCTGTAACT
440     F  E  W  K  V  V  N  P  F  H  V  R  V  R  R  K  N  P  V  T
1378   TCAAACTATGTACACATGGCACTTCAACTGTACCAAGTAGATTATAAATCACACCTCTTG
460     S  N  Y  V  H  M  A  L  Q  L  Y  Q  V  D  Y  K  S  H  L  L
1438   GATTTCAAGATAATATCAAATGATCTTAACGAAGCCTTATATAATGAAAGGAGAAGTACT
480     D  F  K  I  I  S  N  D  L  N  E  A  L  Y  N  E  R  R  S  T
1498   CCAGCTGAGGAAGAAGGTCTTGTTACCACAAGTCACCATGTAATGGAATTCTTTGAAATG
500     P  A  E  E  E  G  L  V  T  T  S  H  H  V  M  E  F  F  E  M
1558   TGTGCAGCTTTAATTACAGAACTAGCACGTTAGatttttgtagagtatatgggcaaataa
520     C  A  A  L  I  T  E  L  A  R  *
1618   gtgttcatacatatctaagctttgatttcttacttcttcaaatggcttcagcaatacatc
1678   tgtattataaaaaaaaaaaaaaaaaaacaaaaaaaaa
```

Figure S2

```
-37     cgtgaactagtggcatataaatacgtcccactgaattATGGGTAACCACACTTCATCAGGA
1                                                M  G  N  H  T  S  S  G
25      GAGCGAAGGGACCGACACAAGTCTGGTGATCCCTATCACTTGGCATCTCCAGGAAGAATT
9         E  R  R  D  R  H  K  S  G  D  P  Y  H  L  A  S  P  G  R  I
85      GATGGACAAGCATTTACTTTTGACAAGCCCAAGGGACAGAAATCATTGCACCAACAGCAC
29        D  G  Q  A  F  T  F  D  K  P  K  G  Q  K  S  L  H  Q  Q  H
145     TCGGAAGAAGACCATGAACCCGTCACTATTAAGCCGTTAAAGCCTAATTCAGATGGGAAA
49        S  E  E  D  H  E  P  V  T  I  K  P  L  K  P  N  S  D  G  K
205     GAGAATGAAGACCCCATGCCAGCTAGGCCTCGGCCAGTCATAACACAGGGGAACAAGAAA
69        E  N  E  D  P  M  P  A  R  P  R  P  V  I  T  Q  G  N  K  K
265     ATGCTCCCGTTTGTCATCAAGTGGACTGGAGGTGGTCAGAAAGTAGCCATTGCTGGTACA
89        M  L  P  F  V  I  K  W  T  G  G  G  Q  K  V  A  I  A  G  T
325     TTCAACGATTGGTTGCAGATTCCAATGGTTAAGAGTGAGAAAGACTTCATAGCCATCGTT
109       F  N  D  W  L  Q  I  P  M  V  K  S  E  K  D  F  I  A  I  V
385     GATTTGCCCGAGGGTCATCACGAGTACAAATTCTATGTTGATGGAGAATGGAAAGTGAGC
129       D  L  P  E  G  H  H  E  Y  K  F  Y  V  D  G  E  W  K  V  S
445     ACTGACGAAGCTTCTTGCGACAACAACATGGGCACCAAAAACAACATCATCACAATCAAA
149       T  D  E  A  S  C  D  N  N  M  G  T  K  N  N  I  I  T  I  K
505     GACCAAGATTTTGAGGAGTTCGAGAATGCTCTTTTGCGTGACCCCAATGACAAGAATAAG
169       D  Q  D  F  E  E  F  E  N  A  L  L  R  D  P  N  D  K  N  K
565     ATAGAACCTAGCAGAATAATCATTAGAGATGAAGAAAAGAAAGATGATTTTAGTCAAGAC
189       I  E  P  S  R  I  I  I  R  D  E  E  K  K  D  D  F  S  Q  D
625     ATCCCCGAATACCAGCAGCTGGAGAAAATACGAGGGCCTCCAGTTCTGCCACCACATCTT
209       I  P  E  Y  Q  Q  L  E  K  I  R  G  P  P  V  L  P  P  H  L
685     CTGCAAGTTATTTTGAATAAGGATACTCCAATTTCATGTGAGCCAACTCTTCTCCCAGAA
229       L  Q  V  I  L  N  K  D  T  P  I  S  C  E  P  T  L  L  P  E
745     CCAAATCACGTTATGTTGAACCACATGTACGCTCTTAGTATTAGAGATGGAATGATGGTT
249       P  N  H  V  M  L  N  H  M  Y  A  L  S  I  R  D  G  M  M  V
805     CTGTCCACGTCTCACCGTTATCGCAAGAAATGTGTCACAACTCTAATCTACAGACCCATT
269       L  S  T  S  H  R  Y  R  K  K  C  V  T  T  L  I  Y  R  P  I
865     GAATAGgccttggatttaggagatagaggctaccagcttattgttacatgccacgtcttt
289       E  *
925     ggatgactcgaaccatttctctttggatgttaaaactcttttttttttctattgctacaa
985     gtccttcatgcctcgatggctgataagtgatatttatattatatattgaatcctgcaatt
1045    atgaaaggcatgcttatgtgttgagaagggccacaaatttgttgacattgtaacggctac
1105    atatcgtctcgtagggtgtaggtgcttcataaaagccgttcattttgtcaagagtttgga
1165    aaaaaggttgttgtcttagttgttggttatggaactatcaaagtggtagcctttatgtaa
1225    gttgtagtgcctttttttcttggcctattctttcattcttaccaattaggtttttttccac
1285    ttaccatcagcctaatgatggaaaactttatagtgttttaagtttctcactgtctttaaa
1345    tatagcagagcttgctgtttatgtggaagtgcaatccaaatgagtcatctgggacttatt
1405    gaagtattatcataaacttttagaagttagatcgcacattattcttgtgcacaaggaaaa
1465    atactgtactctagagtctagaccgttgaagtgacagataaggcatcatgagtctttgca
1525    ttttgggattcttgttcaagcagaaatgcctaaggatttgttcataaatccaactttgtt
```

1585 tgaagtatggtagcttactatttcgttgtgagttttgcttgcaatttttatacatagtag
1645 tgtatagatttttttcattgcattttggtaatttcattttagatataggttttgctttgta
1705 gcctggagctatcattgtggacgtttattgcgcaaaatgttggtattaacgaggtgggag
1765 tcctcatttcttatgtagcaacgttagtcccttacatcccatgagagctagttcttcatc
1825 gcttgaggcttgacatcctgagactggccctcccccccctccacaaaagttatatagaat
1885 ttatataaaattgctcattcctttcttaatccctcatgccagtgctgaacaagatattgt
1945 acatttacgatgtattatggttgatgcctctattgagaatggggacattgcgatagtcct
2005 agaagtcactcagcattttctacttgaatgtcaaggaagactgtatatagggaaaggaag
2065 catatgcattataaaacaagcatttttaatacatgtataattgtacagtagaatgtactg
2125 gaaatgtgtgctttatatgtgttgtatacatgtaaaatatatatatacatatacacatat
2185 tatatacgcagtagataagcctaacactgttcattgtgaaatctgtaaaatctagcttca
2245 aaatcatatttgttctatatgggcagtgcccatatgttggcagagattgagagcagggtg
2305 aagtgatgaactcaatacaagacattgacattactggaatacagtacatatatcatttac
2365 attaatcttgaggtatggctgtattaatattaaaacataggaatacttgtacatttatac
2425 tatatacagtactttcaaacaggaattaactgtaccctacttttaaactttaaacctaac
2485 aaattccagaggtacaaaaaatgttcaacttaatcattcagtttactacagctatttaaa
2545 aagatgtttatagtgcacagggctttactgtactatacccataaaaaagacaaaatttag
2605 ttatacgtattacctttgtacccaatataaattctttagaaaggtaacctttgttagatg
2665 catctattttgaaagtaggagacatccgtaaatgtaaagtgctgtacaattctattaaca
2725 tttaatacatttcaaaatatgaaaatagactttaacatgtgttgaatacaagacacaaat
2785 aaccagacaaatttcatgaaaagagtacagaacacactatgaaatacgatgttaaaacca
2845 ttttcaaaagtaacccacgccattgtggggtagatcatttatttttttcagttacgtaggt
2905 gtttttcatactgtgattatttccgaaaaaatgtgcttgattgaagcttttaattcaaat
2965 gtttaggatttttttttaatattgtatgtttcattcttttcctttgctgtttgtgtatata
3025 ttttacacataagcctattagtgctatatttttgtaacaagctttatgttgcggagaaaa
3085 taaaagacatagactatattgtaagaaaaaaaaaaaaaaaaaaaaaaaaaaa

Figure S3

-54 aggtagcgtgaaggtgtgcctcttagtgtgctcgctggtgagctgtgggacgtcATGGAT

1 M D

7 GGTCCAGACAGCCCACCCTTTGCAGGGCATCCTCACGTCCCCATTATCTGTACTCCAGAC

3 G P D S P P F A G H P H V P I I C T P D

67 GTGGCGCCGGATGATAAGGAGCACAATGGCCTGGCATCCGACAATCCTTTTCCCTTCCCT

23 V A P D D K E H N G L A S D N P F P F P

127 GACGGCATCCCGTCGCCTTACAGGCCGAGCAGCCCTTGTGGAAGCGTCAGGTCCTTTGGC

43 D G I P S P Y R P S S P C G S V R S F G

187 GGCGGGGACAAGGATTCCCACTCGGTGAAGGGGCGGCGGGGCAGCCAGATTCTGCATAAT

63 G G D K D S H S V K G R R G S Q I L H N

247 CTACTGCATCCCCTCGATTACTACCACCACCACCAGCAGAAGAGGCGCAGTCGTGCAAAT

83 L L H P L D Y Y H H H Q Q K R R S R A N

307 TCCATCTCTAGCGACCACGGTAGTGACGTGAACTTAGACCACGAAAAGTCTCCCCCAGTG

103 S I S S D H G S D V N L D H E K S P P V

367 CGAATCCCTTCGCCTCTCATTAGGGTGCCATCCCCGAGGCGCTTCTCCCTGTCCCTCAAA

123 R I P S P L I R V P S P R R F S L S L K

427 TCTGGTCGGTCAAAGACGCCAGATCCTCCGAGGAAGCCCAAAAAGGAAAAGTCTTCGTCC

143 S G R S K T P D P P R K P K K E K S S S

487 CGTCCTGTCACTCCGGAGCCATGGCCAGTTTACCAGCCTGCGGCACGCCCGAGGGCCAGA

163 R P V T P E P W P V Y Q P A A R P R A R

547 CAGAACTTTTTGTGCGTTCCCGACGGTGGATCTGGAATGCCTTCCCCTCTGAGGTATGGG

183 Q N F L C V P D G G S G M P S P L R Y G

607 GGCAGTGGACATAATGTTAATAGTGTACAGAGTAATTCTCGAAGGTATAGTGTCACAGAT

203 G S G H N V N S V Q S N S R R Y S V T D

667 AGTACTTCTAGCAGTAGCTGTAGTGAGAGTAGTTACAGTGACAGTGACGATGAAAACCAG

223 S T S S S S C S E S S Y S D S D D E N Q

727 AGCCCTAGAGAATGGGCCTCTGGTGCCAATAAGGAAAACGAAGATGAGAACCGAAATTAT

243 S P R E W A S G A N K E N E D E N R N Y

787 TCCGCCGACAGTGGGACCGCGAGACCCCCGAACATCCGCATTTCCAGGCCAAAGCCGAGT

263 S A D S G T A R P P N I R I S R P K P S

847 CTCAAACTCAAAATCCCCATTCAAGAAAGTAAGAGCCTGTCTCCCTCTCTCTCTCCGAAC

283 L K L K I P I Q E S K S L S P S L S P N

907 CATTTGCATTCTCCTTATGACCACCTTTTGAGACGAAGATCTCTTTCTAGGTCACCCCAC

303 H L H S P Y D H L L R R R S L S R S P H

967 AGGCCTCTTGTTGATTCTCCGTTGGTGCGAAATGCGTCTCCTGGGCCCGCGCTGAAAAAT

323 R P L V D S P L V R N A S P G P A L K N

1027 TTTGGACACAGTGAACACTTGAGTGCATTTTCTCAATACAGCAGGAGTCCAGGGGACAAT

343 F G H S E H L S A F S Q Y S R S P G D N

1087 ACGCACCACATTTCAAAGGGTAATTTTGTAACAAACATAGAGATCTCTGGGCCGTCGTCG

363 T H H I S K G N F V T N I E I S G P S S

1147 AAGACGTCCATAAACGTTGGGGAAGGGGAGGGATATTCCAGAGGCCTTTTGAGTCCGACT

383 K T S I N V G E G E G Y S R G L L S P T

1207 AGGAAACCGATGGATGATGATGACAACATCAGCTGGGAAAGCTTTTGGGCGGACGACCCG

403 R K P M D D D D N I S W E S F W A D D P
1267 GATGGGAATGCCCACGGCAGTTTTAGGCGGGGTTCCAGGAAGGGTTCTCTTGATAAGGAA
423 D G N A H G S F R R G S R K G S L D K E
1327 TCCTCTGTCGAGAAACTCTACAGCATCTATGATCAGATTATTAAGGAAGGTCAGATGCGC
443 S S V E K L Y S I Y D Q I I K E G Q M R
1387 AGGCACTCTGGGGATGTTGACAGAAGACGACATGGCAGTGGTTCCTCACATCACAATGTA
463 R H S G D V D R R R H G S G S S H H N V
1447 TATGTACGTGGAGAGATGGACCCCAACCAAGCGGCTATACTTTTCCGAGATTCTCGAGGG
483 Y V R G E M D P N Q A A I L F R D S R G
1507 TTACCAGCTGCTGATCCCTTCCTTGAGAACATCAGTAGGAGTGATTTAGAAGATGACGAA
503 L P A A D P F L E N I S R S D L E D D E
1567 TCACAAATATTTGTTAAATTCTTCAAGTTCCACCATACTTACGACCTTATACCACTAAGT
523 S Q I F V K F F K F H H T Y D L I P L S
1627 GCGAAACTTGTGGTATTTGACACTAGGCTGCAGGCGAAAAAGGCTTTCTTTGCCTTGGTA
543 A K L V V F D T R L Q A K K A F F A L V
1687 TACAATGGAGTGCGAGCAGCCCCCCTGTGGGATTCTGCACGTCAGTGCTTCACAGGAATG
563 Y N G V R A A P L W D S A R Q C F T G M
1747 CTCACCATCACAGACTTCATCCGTATTTTACAAAACTTTTATCAGTCACCCAACAGAAAA
583 L T I T D F I R I L Q N F Y Q S P N R K
1807 ATGGAAGAGTTGGAGGATCACCGGTTAGATACTTGGAGAGCCGTATTAAAAGATGAAGAT
603 M E E L E D H R L D T W R A V L K D E D
1867 CGACCGCTGATCAGCATCCGACCCGATGAATCCCTTTATGTTGCAATTCGCTCACTTATT
623 R P L I S I R P D E S L Y V A I R S L I
1927 CATCACAAGATTCATAGATTGCCTGTAATTGACCCTGTCACTGGAAATGTTCTGTACATC
643 H H K I H R L P V I D P V T G N V L Y I
1987 GTTACTCACAAGCGTATCCTCAAGTTCCTTTACTTATATATTAACGAGTTGCCCAAACCG
663 V T H K R I L K F L Y L Y I N E L P K P
2047 TCCATGCTGCAGCAACCTTTAAGAGATCTTGGCATTGGCACCTATGACAAAATTGAAACA
683 S M L Q Q P L R D L G I G T Y D K I E T
2107 GCTAGTCAAGATACACTCATCATTGAAGCTCTTAACAAATTCGTCGAGCACAGAATATCT
703 A S Q D T L I I E A L N K F V E H R I S
2167 GCGCTGCCAATTGTGGATGCACAGGGAAAACTTGTTGACATTTATGCCAAGTTCGATGTC
723 A L P I V D A Q G K L V D I Y A K F D V
2227 ATTAATCTTGCTGCTGAAGGTACATACAACAATCTTGACATCACTCTGCGCAAGGCAAAT
743 I N L A A E G T Y N N L D I T L R K A N
2287 GAATATAGGAATGAGTGGTTTGAGCAGGTACACAAATGTACTCTAGACGAAACATTAGGA
763 E Y R N E W F E Q V H K C T L D E T L G
2347 ACCATAATGGAACGAATAGTTCGAGCTGAAGTTCATCGTTTGGTGGTGGTAGACTCCGAC
783 T I M E R I V R A E V H R L V V V D S D
2407 GACAAAGTGATTGGTGTTATATCCCTCTCGGACATTCTCAAGTACCTTGTACTTAAGCCT
803 D K V I G V I S L S D I L K Y L V L K P
2467 TGCCATGATGTTGAACCCAACAAACTTTCATCTGCCACAGTGACTCAGATGGAGGTCACT
823 C H D V E P N K L S S A T V T Q M E V T
2527 CTTACAGAATCAGAGTCCTCCAATTCGTCAGCAGTTGATGGAAAAGAAACTGTTCCACCA

843 L T E S E S S N S S A V D G K E T V P P
2587 ACTTACCAACCCATGGATACTTCGGCTGAGGATGTTCCATTAAGTGGCCAGTCAGATGTC
863 T Y Q P M D T S A E D V P L S G Q S D V
2647 ACTCCTCCTGATGTCAGTGTAACAAGTGTTGGCAAAACAGTGAGTGAAGATAGTGTTGCA
883 T P P D V S V T S V G K T V S E D S V A
2707 GACAAGTGGAGCAGTGACGACCGCTTGAGTCAGCATGACAGCAGTAGTGGTCGGGGTGAC
903 D K W S S D D R L S Q H D S S S G R G D
2767 AAAAGTGCAGCGAAGGCATCTCCTGCAGATAGTGAGGAGGATGAAGGGCGTTACAGCATG
923 K S A A K A S P A D S E E D E G R Y S M
2827 GGTGATGCAGACGACCCTCCCTCAGTGCCATCCTCTGAGGTTATTCCCATAACGGGGTGA
943 G D A D D P P S V P S S E V I P I T G *
2887 aatgatttcaaagaaaatgttgatttcagtgaactttgatttatacagtatactatataa
2947 gcctcatgcaatacacttgcttcagaaatattgagaacagcatccaaattgttttaacat
3007 aatatattcaggtagcttgtcatgtcatgtccatggttattggtatgatacagtgcagta
3067 gtagtgtgactactttgggtttatttcatcaaaagtattaagtatttttattgttggttc
3127 tacgtggaaaatcatcgaaaagatggcagaggtcaaagtgtggccaataaggtttacata
3187 atactgatctctaattagggcataagccgagaagatggtaagagaagtagaatcaacata
3247 gttcttgcggaagctgtactttgggtgtgtcattgcattatttgatggctatatctaacc
3307 cacatgtaatattaaattggcagttggagctcatcatgtcatgtaatttgatgtccaagt
3367 atatgctgaaaaacaatgttgcaatgccctttttaggttatttatgactggttaacattt
3427 aactgataattccacaggatgaaagtgaaagatatacatatatatattttattagcacgc
3487 agtacaagtgcttcttacagatattgcaagttacttttctcaagggtaatattacaagct
3547 ttactaatgcttatttcattggcaatacaaaagcctgtgcagatatacatagcacacttg
3607 tggcagttggagctcatcatgtcttggcagttggagctcatcatgtcaggctttatatct
3667 tgtttatgaaaaataaagtggaatcgaaaaaaaaaaaaaaaaaaaaaaaaaaa
