# Supplementary material for: Hypoxia Induces Changes in AMP-Activated Protein Kinase Activity and Energy Metabolism in Muscle Tissue of the Oriental River Prawn Macrobrachium nipponense
Source: Front Physiol. 2018 Jun 14;9:751. doi: 10.3389/fphys.2018.00751 (PMC6011032; doi:10.3389/fphys.2018.00751)
Supplement: FIGURE S4 — Amino acid alignment of AMPKα from M. nipponense with ten other known sequences of the AMPKα subunit: Litopenaeus vannamei (AKE50479.1), Homo sapiens (NP_006243.2), Rattus norvegicus (NP_076481.1), Bos taurus (NP_001192534.1), Charadrius vociferous (KGL92907.1), Danio rerio (NP_001103756.1), Drosophila melanogaster (NP_726730.1), Aedes aegypti (XP_001652572.1), Cancer irroratus (ACL13568.1), and Artemia franciscana (ABI13783.1). [file Data_Sheet_2.PDF]

Figure S4

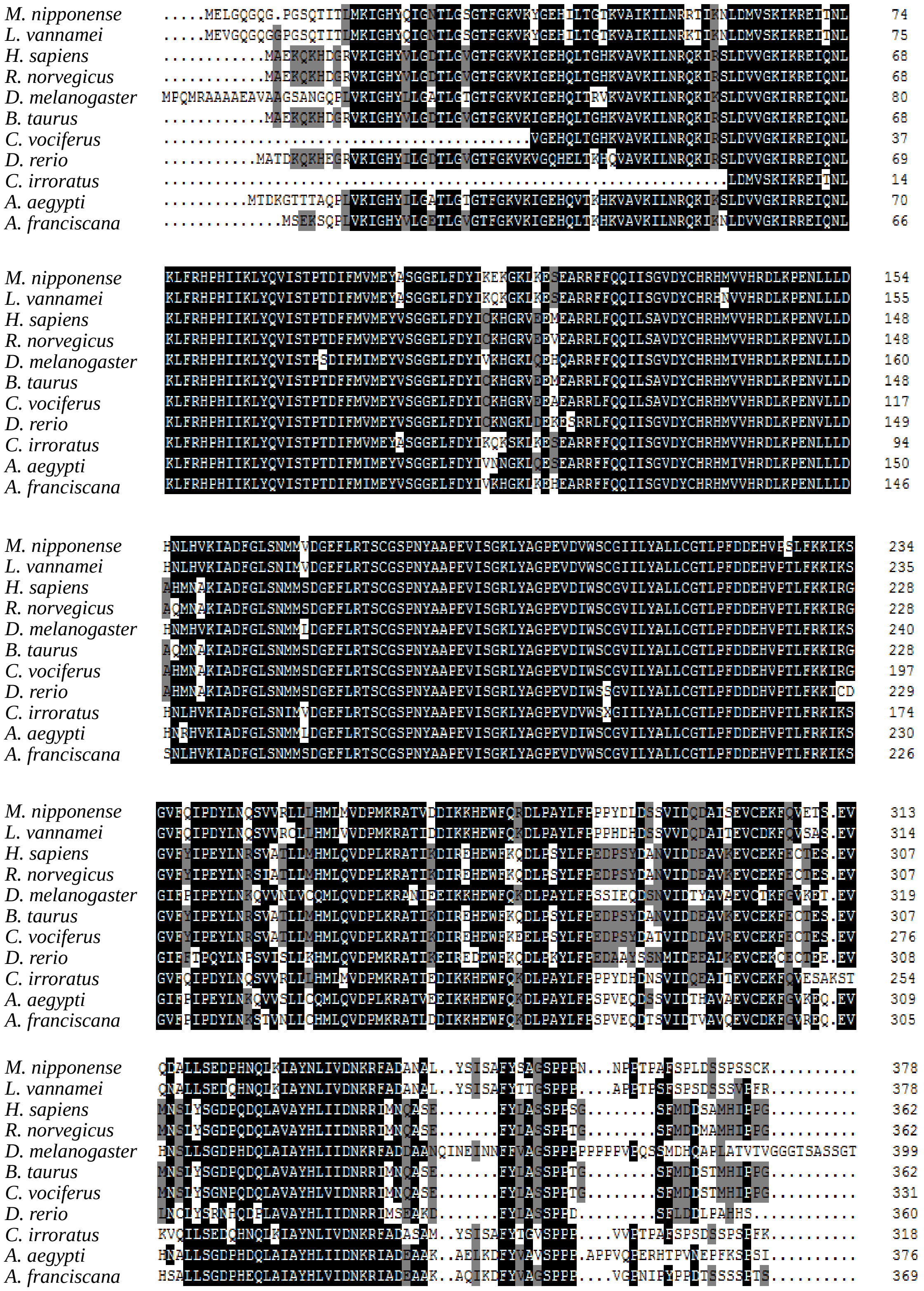
M. nipponense
L. vannamei
H. sapiens
R. norvegicus
D. melanogaster
B. taurus
C. vociferus
D. rerio
C. irroratus
A. aegypti
A. franciscana

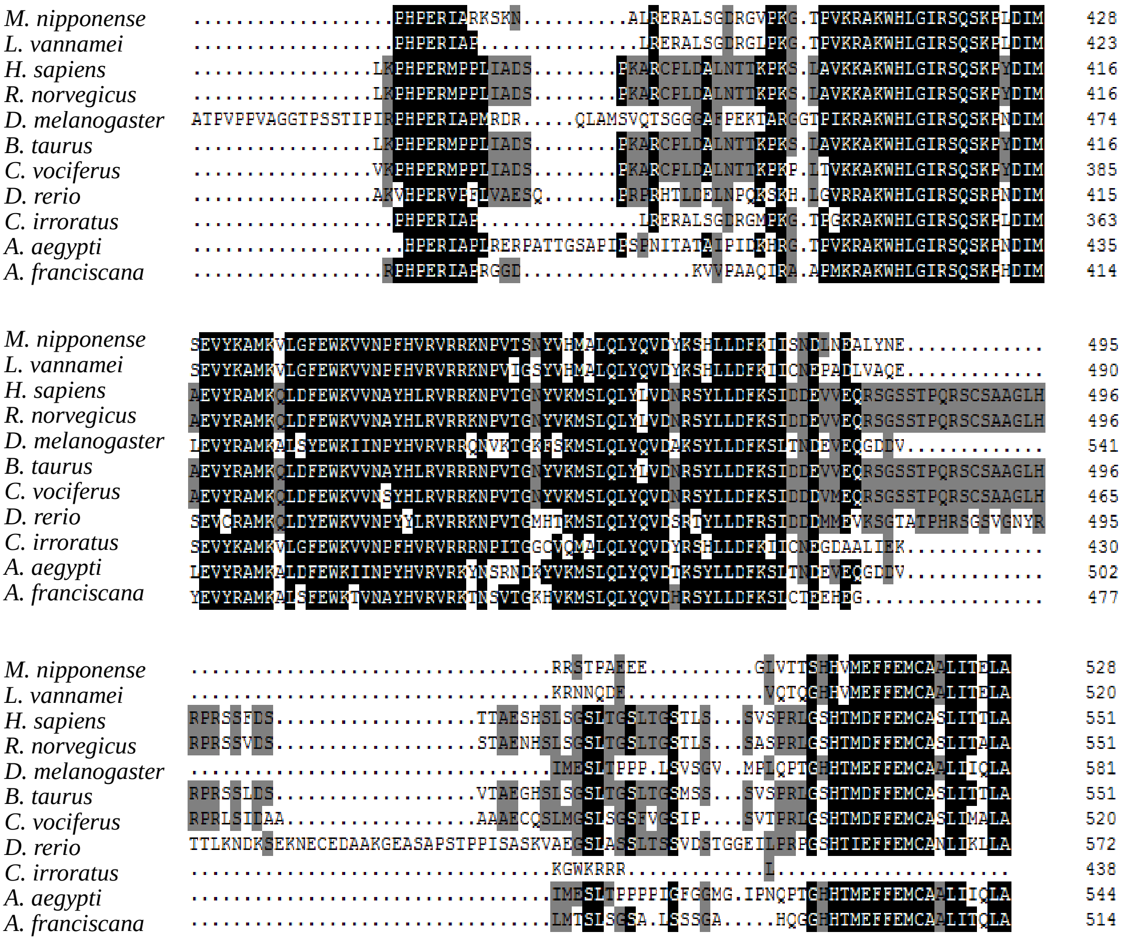

M. nipponense ..................PHPERIARKSKN..........ALRERALSGDRGVPKG.TPVKRAKWHLGIRSQSKPLDIM 428
L. vannamei ..................PHPERIAP...............IRERALSGDRGLPKG.TPVKRAKWHLGIRSQSKPLDIM 423
H. sapiens ................LKPHPERMPPLIADS........PKARCPLDALNTTKPKS.LAVKKAKWHLGIRSQSKPYDIM 416
R. norvegicus ................LKPHPERMPPLIADS........PKARCPLDALNTTKPKS.LAVKKAKWHLGIRSQSKPYDIM 416
D. melanogaster ATPVPPVAGGTPSSTIPIRPHPERIAPMRDR.....QLAMSVQTSGGGAPPEKTARGGTPIKRAKWHLGIRSQSKPNDIM 474
B. taurus ................LKPHPERMPPLIADS........PKARCPLDALNTTKPKS.LAVKKAKWHLGIRSQSKPYDIM 416
C. vociferus ................VKPHPERMPPLIADS........PKARCPLDALNTTKPKP.ITVKKAKWHLGIRSQSKPYDIM 385
D. rerio ................AKVHPERVEPLVAESQ.......PREPHTLDELNPQKSKH.LGVRRAKWHLGIRSQSRPNDIM 415
C. irroratus ..................PHPERIAP...............IRERALSGDRGMPKG.TPGKRAKWHLGIRSQSKPLDIM 363
A. aegypti ...................HPERIAPIRERPATTGSAPIESPNITATAIPIDKHRG.TPVKRAKWHLGIRSQSKPNDIM 435
A. franciscana .................RPHPERIAPRGGD...............KVVPAAQIRA.APMKRAKWHLGIRSQSKPHDIM 414
M. nipponense SEVYKAMKVLGFEWKVVNPFHVRVRRKNPVTSNYVHMALQLYQVDYKSHLLDFKIISNDINEALYNE............ 495
L. vannamei SEVYKAMKVLGFEWKVVNPFHVRVRRKNPVIGSYVHMALQLYQVDYKSHLLDFKIICNEPADLVAQE............ 490
H. sapiens AEVYRAMKQLDFEWKVVNAYHLRVRRKNPVTGNYVKMSLQLYLVDNRSYLLDFKSIDDEVVEQRSGSSTPQRSCSAAGLH 496
R. norvegicus AEVYRAMKQLDFEWKVVNAYHLRVRRKNPVTGNYVKMSLQLYLVDNRSYLLDFKSIDDEVVEQRSGSSTPQRSCSAAGLH 496
D. melanogaster LEVYRAMKALSYEWKIINPYHVRVRRQNVKTGKFSKMSLQLYQVDAKSYLLDFKSLTNDEVEQGDDV............ 541
B. taurus AEVYRAMKQLDFEWKVVNAYHLRVRRRNPVTGNYVKMSLQLYLVDNRSYLLDFKSIDDEVVEQRSGSSTPQRSCSAAGLH 496
C. vociferus AEVYRAMKQLDFEWKVVNSYHLRVRRKNPVTGNYVKMSLQLYQVDNRSYLLDFKSIDDDVLEQRSGSSTPQRSCSAAGLH 465
D. rerio SEVCRAMKQLDYEWKVVNPYYLRVRRKNPVTGMHTKMSLQLYQVDSRTYLLDFRSIDDDMMEVKSGTATPHRSGSVGNYR 495
C. irroratus SEVYKAMKVLGFEWKVVNPFHVRVRRRNPITGGQVQMALQLYQVDYRSHLLDFKIICNEGDAALIEK............ 430
A. aegypti LEVYRAMKALDFEWKIINPYHVRVRKYNSRNDKYVKMSLQLYQVDTKSYLLDFKSLTNDEVEQGDDV............ 502
A. franciscana YEVYRAMKALSFEWKTVNAYHVRVRKTNSVTGKHVKMSLQLYQVDHRSYLLDFKSLCTEEHEG................ 477
M. nipponense ....RRSTPAEEE..........GLVTTSHHVMEFFEMCAALITELA 528
L. vannamei ....KRNNQDE............VQTQGHHVMEFFEMCAALITELA 520
H. sapiens RPRSSFDS..................TTAESHSLSGSLTGSLTGSTLS...SVSPRLGSHTMDFFEMCASLITTLA 551
R. norvegicus RPRSSVDS..................STAENHSLSGSLTGSLTGSTLS...SASPRLGSHTMDFFEMCASLITALA 551
D. melanogaster ...............................IMESLTPPP.LSVSGV..MPLQPTGHHTMEFFEMCAALIIQLA 581
B. taurus RPRSSLDS..................VTAEGHSLSGSLTGSLTGSMSS...SVSPRLGSHTMDFFEMCASLITTLA 551
C. vociferus RPRLSIDAA.................AAAECQSLMGSLSGSFVGSIP....SVTPRLGSHTMDFFEMCASLIMALA 520
D. rerio TTLKNDKSEKNECEDAAKGEASAPSTPPISASKVAEGSIASSLTSSVDSTGGEILPRPGSHTIEFFEMCANLIKILA 572
C. irroratus ...............................KGWKRRR.............I...................... 438
A. aegypti ...............................IMESLTPPPPIGFGGMG.IPNQPTGHHTMEFFEMCAALIIQLA 544
A. franciscana ...............................LMTSLSGSA.LSSSGA.....HQGGHHTMEFFEMCAALITQLA 514

Figure S5

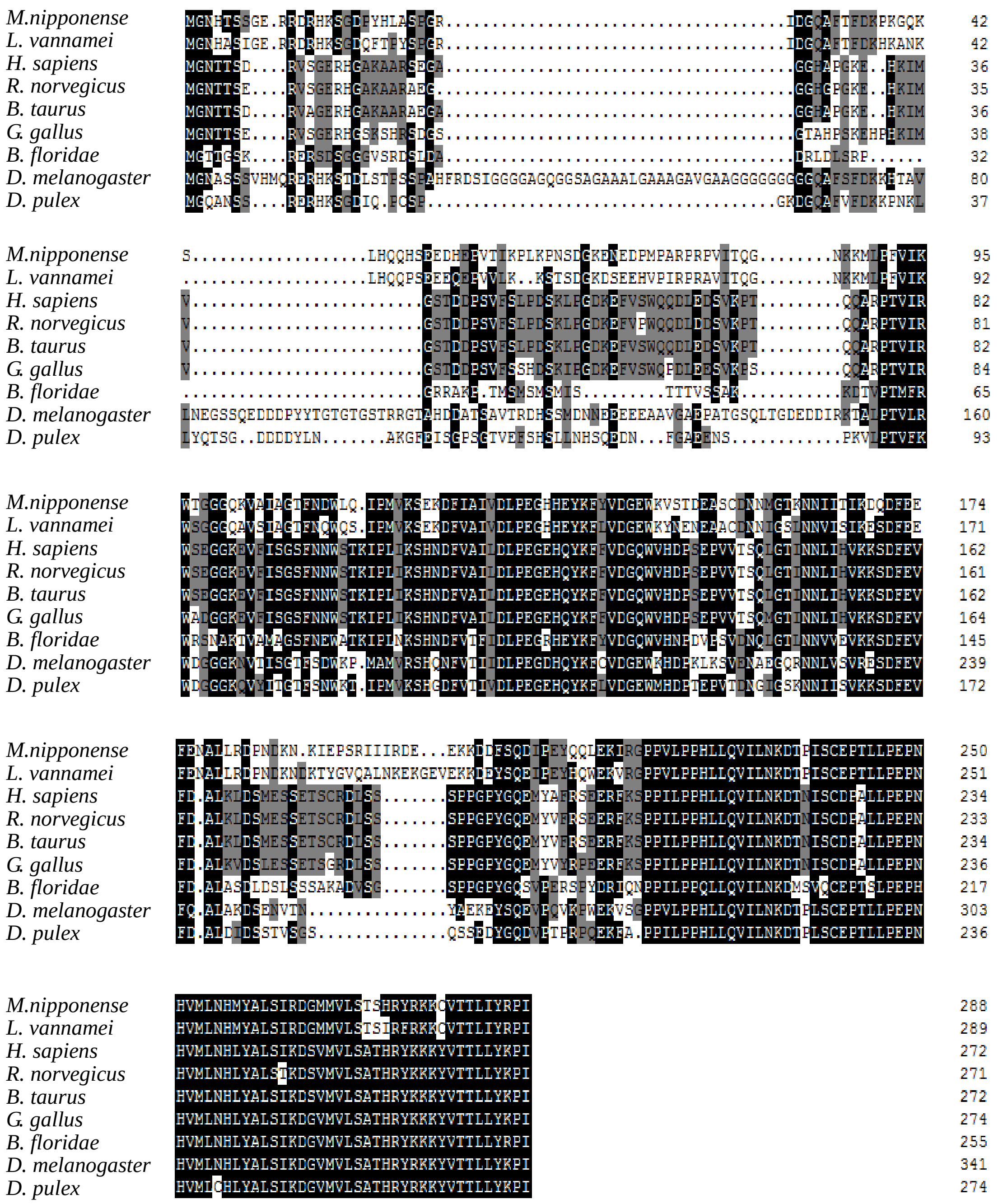
M.nipponense
L. vannamei
H. sapiens
R. norvegicus
B. taurus
G. gallus
B. floridae
D. melanogaster
D. pulex

Figure S6

```
M. nipponense    MDGPDSPPFAGHPHVPIICTPDVAPDDKEHNGLASDNPFPFPDGIPSPYRPSSPCGSVRSFGGGDKDSHSVKGRRGSQIL    80
L. vannamei      MDGASSPVFTGFPLPPIIFTPDMSPRDHESS..PMDVPGAVEHG.ASGNKSGASSPGV.DFNSINSKEGSVKGKGGA.LL    75
H. sapiens       ................................................................................     0
R. norvegicus    ................................................................................     0
B. taurus        ................................................................................     0
C. canorus       ................................................................................     0
B. mori          ................................................................................     0
S. salar         ................................................................................     0
D. melanogaster  MHG.ITHLYRQHAVEKQLSGGESWSRQIYPSYGSSADASSQGSRLYSVDSSSSSSSNSNNSGGGGSGTAGVENLGGV...    76
A. aegypti       ................................................................................     0
C. irroratus     ................................................................................     0
H. americanus    ................................................................................     0
C. maenas        ................................................................................     0

M. nipponense    HNLLHPLDYYHHHQQKRRSRANSISSDHGSDVNLDHEKSPPVRIPSPLIRVPSPRRFSLSLKSGRSKTPDPPRKPKKEKS   160
L. vannamei      HHFLHPLDYYHQHQAKRRSRNNSISSDHG.DITLELANAHAQ...........AQANKEKEKSFRSKTPDPPRVQRQERE   143
H. sapiens       ................................................................................     0
R. norvegicus    ................................................................................     0
B. taurus        ................................................................................     0
C. canorus       ................................................................................     0
B. mori          ................................................................................     0
S. salar         ................................................................................     0
D. melanogaster  .....TGSAYAQWQSDRHSLPQAVPDAPRSLANHHYRNSPTH............QAHYQQPTSGSLKRTPSSKRSFLERS   139
A. aegypti       ......................MGDSADGGTQFQRPSTAPAG...............TDSARSSKRMKKSEREAKKQEKE    43
C. irroratus     ................................................................................     0
H. americanus    ................................................................................     0
C. maenas        ................................................................................     0

M. nipponense    SSRPVTPEPWPVYQPAAR..PRARQNFLCVPD.....GGSGMPSPLRYGGSGHNVNSVQSNSRRYSVTDSTSSSSCSESS   233
L. vannamei      RSR..TPEPWPVYRPQARQRPRPRNLFIPVPNQPLSKGSPVVTSPTKSEGSYYQRRGSGAESIPSIASSESGSSTGSSGS   221
H. sapiens       ..............................................................MPLLDGDLEGSGKHSSR.    17
R. norvegicus    ................................................................................     0
B. taurus        ................................................................................     0
C. canorus       ........................................................DLSSFAMPFLDGDVETTDKNASR.    23
B. mori          ....................................................METRTSSSSHHYHTHHADS...RRR...    22
S. salar         ................................................................................     0
D. melanogaster  HSP.......AIYGSMRR...SAGPDFGSPPPTAPVGGGGYFP......HDLDDIYESQTDHQYFTHTGASGPQQQRPTA   203
A. aegypti       QKK........IERETARRLEREAAKLEK......................LNRKHENISRSTERVSGGRSGSLERRRS.    92
C. irroratus     ................................................................................     0
H. americanus    ................................................................................     0
C. maenas        ................................................................................     0

M. nipponense    Y....SDSDDENQSPREWASGANKENEDENRNYSADSG..TARPPNIRISRPKPSLKLKIPIQESKSLSPSLSPNHLHSP   307
L. vannamei      YTGSESESESEEEKPKTWSSGVRTLGQEYSQVSSSPGKEQASSPPATSPSIQAKRPSVSLPIDQN...ARPKSPN.FHFP   297
H. sapiens       ..........KVDSPFGPGSPSKGFFSRGPQPRPSSPMSAPVRPKTSP...GSPKTVFPFSYQES............PPR    72
R. norvegicus    ................................................................................     0
B. taurus        ............MEPAELEHALCG.SLFSTQTPSWSSFGGPEHQEMSFLEQGDSTSWPSPAMTTS.............AE    54
C. canorus       ..........KVEDSYSGSSPSKGLFSKGLQNRPSSPVSAPVRSKHSP...GSPKTVFPFPYQES............PPR    78
B. mori          ..........HEGDRYQTSVNTYQTPRNEYRQFVSPLFNDAMDDRYKS.RFDNKNFAQKLTESVS.......SSS..KTT    82
S. salar         ................................................................................     0
D. melanogaster  I......SIFHRVSAAHTSLDDYTLPGDASRQSMNSTDSGVSSGPFNRQRFDNSSFAKSLSIDEQ.......DPQ..HSP   268
A. aegypti       ..........GDDGPVLNQSTVHGIASPNRRPTIFDVFR.PRKGSDSKKKKDDSSKSSSDKDSTS.......GSGTITGS   154
C. irroratus     ................................................................................     0
H. americanus    ................................................................................     0
C. maenas        ................................................................................     0

M. nipponense    YDHLLRRRSLSRSPHRPLVDSPLVRNASPCPALKNFGHSEHL......SAFSQY.....SRSPGDNTHH...........   365
L. vannamei      FEYLYRRSSLSKSSRRGSIESPLAQNSFPCPTSPTETKTDSLKVPKPHSPFNWYRRRSMSKSPNRPPHAPLGQTTSSPTK   377
H. sapiens       SPRRMSFSGIFRSSSKESSPNSNPATSPGCIRFFSRSR..........................................   110
R. norvegicus    ................................................................................     0
B. taurus        ISLGEQRTKVSRWKSQEDVEERELPGLEGCPQSR..............................................    88
C. canorus       SPRRMSFSGIFRSSSKESSPNSNPSTSPGCIKFFSRSR..........................................   116
B. mori          SSE.RRGSGSSGSGHRHKHHSIQELIRTFCKKVGSWRH............................ESG...........   122
S. salar         ................................................................................     0
D. melanogaster  HKEGHSKHGKHHHHHSHHHHSIHELVKHFCKKMHLWPR............................KHH...........   309
A. aegypti       GGIMNSMKAVLHVGGRHSHHTGAAHPASACATKVRDGS..........................AHPHAG..........   198
C. irroratus     ................................................................................     0
H. americanus    ................................................................................     0
C. maenas        ................................................................................     0
```

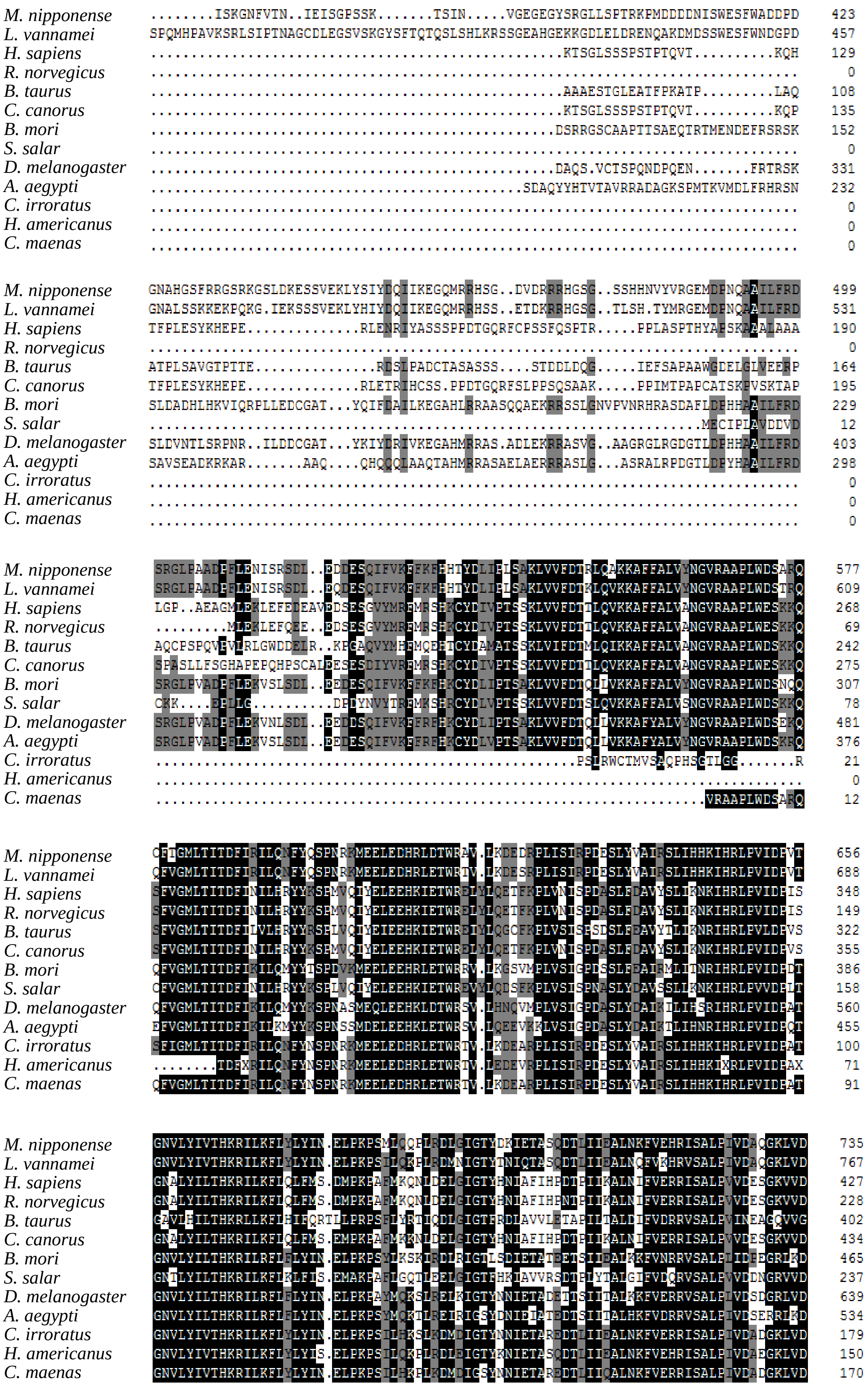
M. nipponense
L. vannamei
H. sapiens
R. norvegicus
B. taurus
C. canorus
B. mori
S. salar
D. melanogaster
A. aegypti
C. irroratus
H. americanus
C. maenas

```
M. nipponense     IYAKFDVINLAAEGTYNNLDITLRKANEYRNEWFEQVHKCTLDETLGTIMERIVRAEVHRLVVVDSDDKVIGVISLSDIL   815
L. vannamei       IYAKFDVINLAAEGTYNNLDVTLRKANEYRNEWFEQVHRCTLDETLETVMERIVRAEVHRLVVVDEEGRVVGVISLSDIL   847
H. sapiens        IYSKFDVINLAAEKTYNNLDITVTQALQHRSQYFEGVVKCNKLEILETIVDRIVRAEVHRLVVVNEADSIVGIISLSDIL   507
R. norvegicus     IYSKFDVINLAAEKTYNNLDITVTQALQHRSQYFEGVVKCSKLETLETIVDRIVRAEVHRLVVVNEADSIVGIISLSDIL   308
B. taurus         LYSRFDVIHLAAQQTYNHLDISVGEALRRRTLCLEGVLSCQPHETLGEVIDRIAREQVHRLVLVDETQHLLGVVSLSDIL   482
C. canorus        IYSKFDVINLAAEKTYNNLDITVTQALQHRSQYFEGVVKCSMLETLETIVDRIVKAEVHRLVVVNEADSIVGIISLSDIL   514
B. mori           IYAKFDVINLAAEKTYNNLDVTLKTANEHRNEWFEGVQKCKLDETLFDVMERIVRAEVHRLVVVDDDDKVIGIISLSDLL   545
S. salar          IYSKFDVINLAAEKMYNNLDVTVTKALQHRSQYFEGVLTCNTHDTLESIINRLVEAEVHRLVVVDEQEVVKGIVSLSDIL   317
D. melanogaster   IYAKFDVINLAAEKTYNDLDVSLRKANEHRNEWFEGVQKCNLDESLYTIMERIVRAEVHRLVVVDENRKVIGIISLSDIL   719
A. aegypti        IYAKFDVINLAAEKTYNDLDVSLKTANEHRNAWFEGVQHCKLDETLYTVMERIVRAEVHRLVVVDEEEKVIGIISLSDIL   614
C. irroratus      IYAKFXV.........................................................................   186
H. americanus     IYAXFDVINLAA....................................................................   162
C. maenas         IYAKFDVIN.......................................................................   179
```

```
M. nipponense     KYLVIKPCHDVEPNKLSSATVTQMEVTLTESESSNSSAVDGKET.VPPTYQPMDTSAEDVPLSG...QSDVTPP..DVSV   889
L. vannamei       KELVIKPCKDTEPNGLQSATVSQMEVTLTTSDLSTSPTKPSAEDGMDPSTEVSQDSSENSPAELNIKEVEVTPPA.DVNI   926
H. sapiens        QALIILTPAGAKQKETETE.............................................................   525
R. norvegicus     QALIILTPAGAKQKETETE.............................................................   326
B. taurus         QALVISPAGIDALGA.................................................................   497
C. canorus        QALVITPAG.......................................................................   523
B. mori           MYLVIRPTGECG...VTSLRNEHAPIEEKD................................ENLNPEDVAESGD.DKST   589
S. salar          QALVITNEEAD.....................................................................   328
D. melanogaster   LYLVIRPSGEGVGGSESSLRASDPVLLRKVAEVEIPATAAAATT.TTPPRSPSAGSGNRSLIEDIPEEETAPARS.DDAD   797
A. aegypti        LYLVIRPTGDGIGN.SESLRATDPRIRKSPNKLSQTKR.........DSNESIEEEKESTPEEAVETEEESNSGGKDTAA   684
C. irroratus      ................................................................................   186
H. americanus     ................................................................................   162
C. maenas         ................................................................................   179
```

```
M. nipponense     TSVGKTVSEDSVADKWSSDDRLSQH.DSSSGRGDKSAAKASPADSEEDEGRYSMGDADDPPSVPSSEVIPITG.......   961
L. vannamei       NVVRKTESEDSVEGKWSSDDRLSQHCSSSSGKVDQDAAKASPADSEEDEGRYSMGDADDPP...QAEVIPITG.......   996
H. sapiens        ................................................................................   525
R. norvegicus     ................................................................................   326
B. taurus         ................................................................................   497
C. canorus        ................................................................................   523
B. mori           VSKEQDDSEERTQCQE................................................................   605
S. salar          ................................................................................   328
D. melanogaster   SDNNKSASEDKANNNQHDQTTTAATANGDSNNSPVEVSFADEAQEEEAADQVERSNCDDDDQPALAEIERKNASMDDDED   877
A. aegypti        NKTEPAEDEEENGDQDQAEDEDVPPTESPADHSSNGSPVQEMLNEEHKAIRDTLASNVQREIGLVSE.............   751
C. irroratus      ................................................................................   186
H. americanus     ................................................................................   162
C. maenas         ................................................................................   179
```

```
M. nipponense     ............................   961
L. vannamei       ............................   996
H. sapiens        ............................   525
R. norvegicus     ............................   326
B. taurus         ............................   497
C. canorus        ............................   523
B. mori           ............................   605
S. salar          ............................   328
D. melanogaster   DGMSSAVSAASALGQSLTPAAQEMALVS   905
A. aegypti        ............................   751
C. irroratus      ............................   186
H. americanus     ............................   162
C. maenas         ............................   179
```
